# ISG15 Enhances the Activity of γ‐Glutamate Cysteine Ligase to Suppress Apoptosis in High Fat Diet‐Promoted Hepatocellular Carcinoma

**DOI:** 10.1002/advs.202416401

**Published:** 2025-03-24

**Authors:** Xinran Liu, Qiujin Ma, Zhao Jia, Yihao Zhou, Churong Zou, Yushuo Xiao, Yuchen Chen, Chuyao Ma, Liangliang Song, Jing Yang, Chen Wang, Huidie Xu, Hong Chen, Jiajian Shi, Junqiu Yue, Yu Sun, Desheng Hu, Robert B Petersen, Yangkai Li, Anlin Peng, Kun Huang, Ling Zheng

**Affiliations:** ^1^ School of Pharmacy Tongji Medical College and State Key Laboratory for Diagnosis and Treatment of Severe Zoonotic Infectious Diseases Huazhong University of Science & Technology Wuhan 430030 China; ^2^ State Key Laboratory of Metabolism and Regulation in Complex Organisms, TaiKang Center for Life and Medical Sciences; Frontier Science Center for Immunology and Metabolism, College of Life Sciences Wuhan University Wuhan 430072 China; ^3^ Department of Pathology Hubei Cancer Hospital Tongji Medical College Huazhong University of Science and Technology Wuhan 430079 China; ^4^ Department of Integrated Traditional Chinese and Western Medicine Union Hospital Tongji Medical College Huazhong University of Science and Technology Wuhan 430000 China; ^5^ China‐Russia Medical Research Center for Stress Immunology Union Hospital Tongji Medical College Huazhong University of Science and Technology Wuhan 430000 China; ^6^ Foundational Sciences Central Michigan University College of Medicine Mt. Pleasant MI 48859 USA; ^7^ Department of Thoracic Surgery Tongji Hospital Tongji Medical College Huazhong University of Science and Technology Wuhan 430030 China; ^8^ Department of Pharmacy The Third Hospital of Wuhan Tongren Hospital of Wuhan University Wuhan 430070 China

**Keywords:** γ‐glutamate cysteine ligase, glutathione, hepatocellular carcinoma, high fat diet, interferon stimulated gene 15, reactive oxygen species

## Abstract

Obesity is a leading risk factor for development of hepatocellular carcinoma (HCC). High‐fat intake produces cytotoxic effects in liver cells, such as excessive reactive oxygen species (ROS) accumulation and apoptosis. How HCC cells regulate ROS level and escape the cytotoxic effects of high fat diet (HFD) stress remains unclear. Herein, this work reports a critical anti‐ROS/apoptotic role of the ubiquitin‐like protein interferon stimulated gene 15 (ISG15) in HFD‐promoted HCC. In mouse models and clinical HCC samples, upregulation of ISG15 is associated with hepatic steatosis. Notably, upregulated ISG15 elevates cellular glutathione levels, which subsequently reduces ROS accumulation and confers resistance to apoptosis in HCC cells. In diethylnitrosamine‐induced HCC mouse model, HFD‐feeding promotes HCC progression in wildtype mice, while tumor growth is significantly suppressed accompanied by apoptosis of HCC cells in *Isg15*‐KO mice. Mechanistically, ISG15 promotes the activity of γ‐glutamate cysteine ligase (γ‐GCL), a rate‐limiting heterodimeric holoenzyme of glutathione synthesis consisting of glutamate‐cysteine ligase catalytic subunit (GCLC) and glutamate‐cysteine ligase modifier subunit (GCLM). Independent of ISGylation, ISG15 forms an ISG15/GCLM/GCLC complex that promotes GCLM‐GCLC interaction, increases glutathione generation and inhibits HFD‐induced apoptosis in HCC cells. Together, an anti‐apoptotic ISG15‐γ‐GCL‐glutothione axis is suggested in HFD‐promoted HCC.

## Graphical abstract

Hepatocellular carcinoma (HCC) cells under high‐fat environment constrain harmful reactive oxygen species (ROS) by enhancing glutathione synthesis though upregulating interferon stimulated gene 15 (ISG15). In this process, upregulated high mobility group A1 (HMGA1) enhances ISG15 transcription, upregulated ISG15 subsequently noncovalently binds with glutamate‐cysteine ligase modifier subunit (GCLM) and increases the GCLM‐glutamate‐cysteine ligase catalytic subunit (GCLC) interaction to form an ISG15/GCLM/GCLC complex, which has increased γ‐glutamate cysteine ligase (γ‐GCL) enzymatic activity that promotes glutathione synthesis to protect HCC cells from ROS‐induced apoptosis.

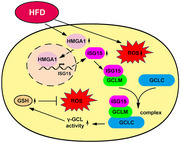

## 1 Introduction

Hepatocellular carcinoma (HCC) constitutes 75–85% of the new cases of primary liver cancer.^[^ ^1^ ^]^ Obesity, caused by unhealthy lifestyles such as overnutrition, is a major risk factor for development of HCC in many regions.^[^ ^2^, ^3^ ^]^ Dietary obesity is the main cause of non‐alcoholic fatty liver disease (NAFLD), which may progress to non‐alcoholic steatohepatitis (NASH), and eventually HCC through a cumulative and complex process.^[^ ^4^ ^]^ Several mechanisms linking obesity to HCC have been identified, including metabolic reprogramming, oxidative stress and inflammation.^[^ ^5^, ^6^, ^7^, ^8^ ^]^

On the other hand, excessive lipid intake and the consequent accumulation of reactive oxygen species (ROS) can cause toxic effects,^[^ ^9^ ^]^ such as apoptosis, to HCC cells.^[^ ^10^ ^]^ In normal cells, anti‐oxidative stress regulators, including nuclear factor erythroid 2‐related factor 2 (NRF2) and tumor suppressor protein p53, are activated to prevent ROS accumulation.^[^ ^9^ ^]^ However, under pathological conditions, dysfunction of ROS regulation often occurs. For examples, NRF2 signaling is suppressed during NAFLD development,^[^ ^11^, ^12^ ^]^ and defects or loss of function mutations in the p53 pathway occurs in more than 50% of HCC patients.^[^ ^13^ ^]^ How HCC cells cope with oxidative stress during NAFLD‐HCC progression remains largely unknown.

As an endogenous ROS scavenger, glutathione performs an essential antioxidant role.^[^ ^14^ ^]^ The level of intracellular glutathione is mainly determined by γ‐glutamate cysteine ligase (γ‐GCL), the rate‐limiting enzyme in glutathione synthesis.^[^ ^15^ ^]^ γ‐GCL has two subunits, the glutamate‐cysteine ligase catalytic subunit (GCLC) contributes to enzymatic activity, and the glutamate‐cysteine ligase modifier subunit (GCLM) modulates holoenzyme formation and enzymatic activity.^[^ ^15^ ^]^ Heterodimerization of GCLM with GCLC forms the γ‐GCL holoenzyme which increases the enzyme activity of GCLC approximately fivefold.^[^ ^15^ ^]^ However, the precise mechanism by which GCLM and GCLC are coordinated to regulate γ‐GCL enzymatic activity remains unclear.

Interferon stimulated gene 15 (ISG15) is a ubiquitin‐like protein. Upon viral infection, Interferon (IFN) regulatory factors (IRFs) control its transcription through stimulator of interferon genes‐interferon regulatory factor 3 (STING‐IRF3) or Janus kinase/signal transducer and activator of transcription‐interferon regulatory factor 3 (JAK/STAT‐IRF3) signaling to protect the host from infection.^[^ ^16^ ^]^ Similar to ubiquitination, ISG15 may be covalently conjugated to substrate proteins, a process known as ISGylation, which involves a three‐step cascade involving the E1 activating enzyme, E2 conjugating enzyme, and E3 ligase.^[^ ^17^ ^]^ ISG15 can also function in an unconjugated intracellular form, for example, it is noncovalently bound to ubiquitin specific peptidase 18 (USP18) to regulate IFN signaling.^[^ ^16^ ^]^ Moreover, unconjugated ISG15 can be secreted by leukocytes and functions as a cytokine,^[^ ^18^ ^]^ signaling lymphocytes to produce immunomodulatory cytokines such as interleukin 10 (IL‐10) and IFN‐γ.^[^ ^18^ ^]^ Accumulating evidence indicates that ISG15 is upregulated in cancers, suggesting that it is an oncoprotein in a number of solid tumors.^[^ ^19^, ^20^, ^21^ ^]^ However, the function of ISG15 in cancer at a molecular level is not fully understood.

Herein, we report that ISG15 confers resistance to apoptosis in HCC cells by regulating glutathione production. High fat diet increases ISG15 expression by upregulating high mobility group A1 (HMGA1) in HCC cells, and upregulated ISG15 noncovalently binds with the GCLM subunit to enhance GCLM‐GCLC interaction, which subsequently increases glutathione production to reduce ROS and inhibit apoptosis of HCC cells. This ISG15‐γ‐GCL‐glutathione axis suggests a novel antioxidative pathway used by HCC cells to prevent HFD induced cytotoxicity.

## 2 Results

### 2.1 High Fat Diet Promotes Hepatic ISG15 Expression in DEN‐Induced HCC Mice

**Figure 1:**
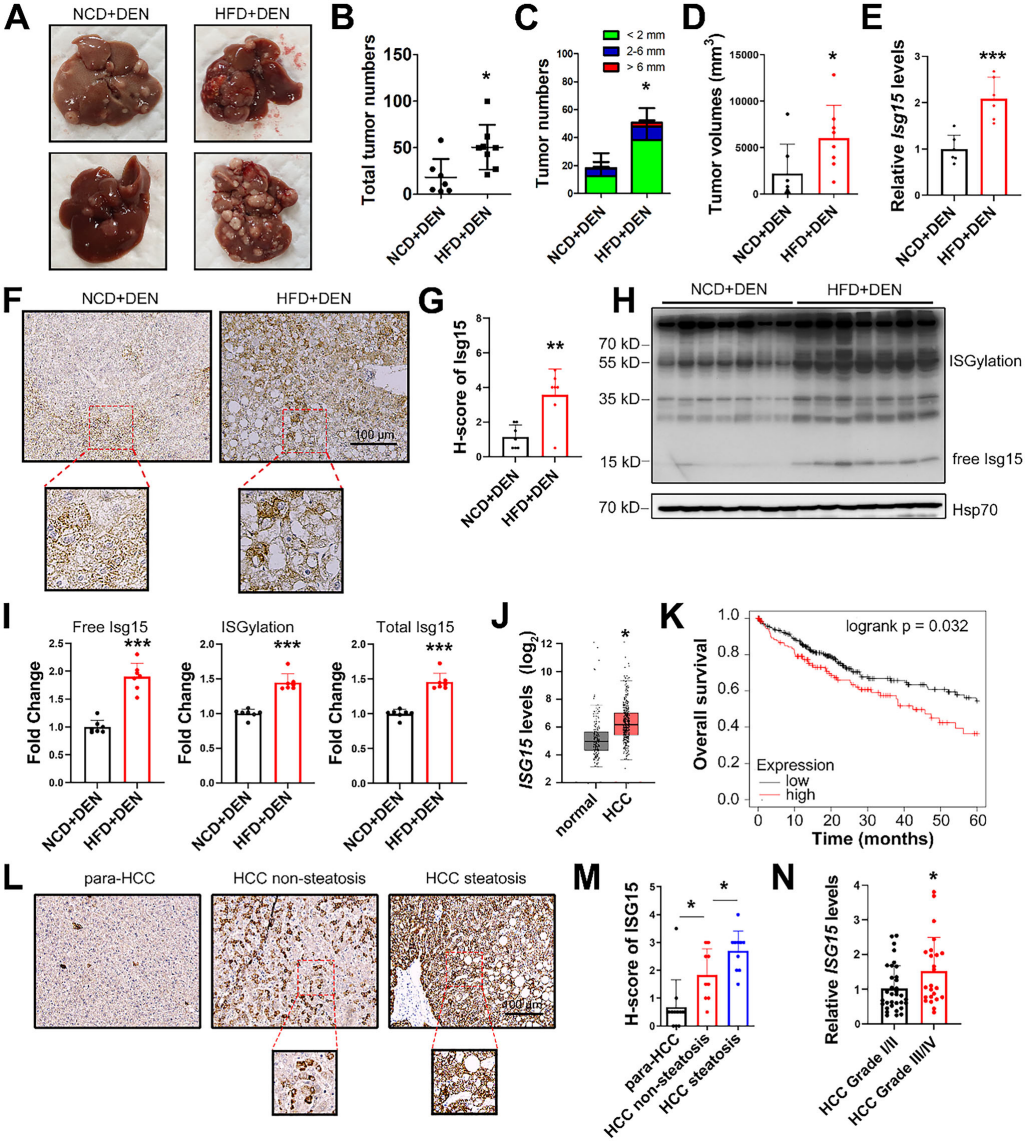
ISG15 is upregulated in HCC under fat challenge. A) Representative liver images for the indicated groups. B) Total tumor numbers. C) Numbers of tumors with different diameters. D) Quantification of total tumor volumes. E) The mRNA levels of *Isg15* in mice HCC tissues. F,G) Representative images (F) and H‐score quantification (G) of Isg15 IHC staining in murine HCC tissues. H,I) Western blots (H) and the relative integrated density values (I) of free, conjugated forms and total Isg15 in murine HCC tissues. J) mRNA levels of ISG15 in HCC and para‐HCC tissues. Data from GEPIA. K) Over‐all survival rates of HCC patients with high ISG15 expression levels (red) and low levels (black). Data from UALCAN. L,M) Representative IHC images (L) and H‐score quantification (M) of ISG15 in human HCC clinical samples. N) The mRNA levels of *ISG15* in clinical HCC samples of indicated grades. RNA‐seq data was from GSE195952. Data shown as mean ± SD. *p < 0.05; **p < 0.01; ***p < 0.001.

To study the effect of HFD on HCC development, mice were injected intraperitoneally with DEN 14 days after birth, then were fed with normal chow diet (NCD) or HFD until sacrifice at age of 40 weeks. The results suggested that HFD significantly promoted HCC progression (**Figure** **1**A–D). To investigate how obesity promotes HCC, by using our previously reported RNA sequencing data (GEO GSE117539),^[^ ^8^ ^]^ we compared differentially expressed hepatic genes in DEN‐induced HCC mice fed with HFD with those fed with NCD. We noticed that the level of Isg15, which plays an important role in cancer,^[^ ^22^ ^]^ was upregulated in HCC tumors from HFD‐fed mice than in those from NCD‐fed mice (Figure 1E–G). Western blots demonstrated elevated levels of all forms of Isg15 (conjugated, free, and total) in HCC tumors from HFD‐fed mice (Figure 1H,I).

Upregulation of ISG15 was also found in clinical samples from HCC patients. Analysis of 369 HCC and 160 non‐tumor samples from the GEPIA database demonstrated a significant upregulation of ISG15 in HCC tissues (Figure 1J). Moreover, the survival rates of HCC patients were lower in HCC patients with higher‐than‐average ISG15 levels (p = 0.032, Figure 1K, data from UALCAN database). To investigate the clinical relevance of the ISG15 level and steatosis in HCC, clinical samples (10 fatty liver‐associated HCC, and 10 non‐fatty liver‐associated HCC) were studied. An increase in ISG15 was found in pathological sections from HCC tissues with steatosis compared with those from steatosis‐free HCC (Figure 1L,M). Moreover, *ISG15* levels in HCC of different grades and metastatic samples were also studied (GSE195952 & GSE63018),^[^ ^23^, ^24^ ^]^ elevated *ISG15* was found in low differentiated HCC tissues (grade III/IV) compared with high differentiated HCC tissues (graded I/II, Figure 1N), whereas no significant difference was found between the primary HCC and metastatic tumor samples (Figure S1A, Supporting Information). In patients of early HCC stage (stage I/II), the overall survival rates were obviously lower in patients with higher‐than‐average ISG15 levels (*p* = 0.0064, Figure S1B, Supporting Information, data from cBioPortal database); whereas in patients with advanced HCC stage (stage III/IV), the difference was not significant (*p* = 0.15, Figure S1B, Supporting Information).

Excessive lipid deposition often leads to pathology of organs, such as liver and kidney.^[^ ^25^, ^26^ ^]^ To determine whether ISG15 is upregulated in other fat‐associated diseases, we studied ISG15 transcriptional levels in NAFLD (hepatic steatosis) and minimal change glomerulopathy (MCD) (GEO GSE130970 & GSE200828).^[^ ^27^ ^]^ Significantly elevated ISG15 was found in hepatic steatosis and MCD compared with normal liver and kidney tissues (Figure S1C,D, Supporting Information).

### 2.2 HMGA1 is Involved in Palmitic Acid‐Induced ISG15 Upregulation

**Figure 2:**
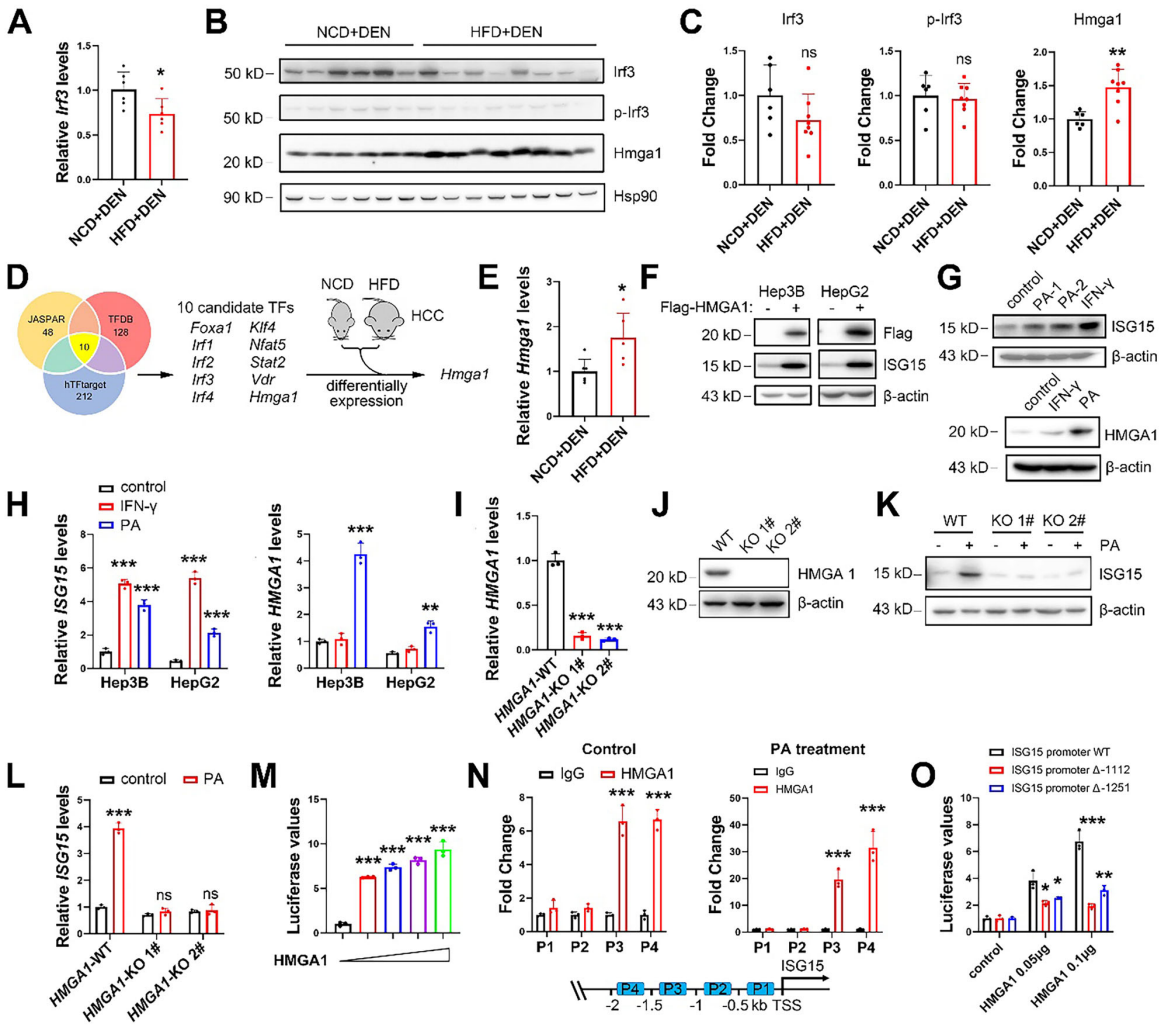
PA transcriptionally upregulates ISG15 through HMGA1. A) mRNA levels of *Irf3* in murine HCC tissues. B,C) Western blots (B) and the relative integrated density values (C) of Hmga1, Irf3 and phosphorylated Irf3 in murine HCC tissues. D) Potential transcription factor (TF) candidates for *Isg15* under HFD challenge screened through JASPAR, TFDB and hTFtarget databases. Ten candidates were found in RNA‐seq data to detect differently expressed genes in HFD versus NCD samples, and *Hmga1* was identified. E) mRNA levels of *Hmga1* in murine HCC tissues. F) Western blots of endogenous ISG15 in Hep3B and HepG2 cells at 24 h after transfection of the plasmid expressing Flag‐HMGA1. G) Western blots of ISG15 (top) and HMGA1 (bottom) in Hep3B cells under PA (100 µM) or IFN‐γ treatments for 24 h. H) mRNA levels of *ISG15* and *HMGA1* in Hep3B and HepG2 cells under PA (100 µM) or IFN‐γ treatment for 24 h. Compared with the control group, the *ISG15* levels were significantly elevated both by PA and IFN‐γ treatments; whereas the *HMGA1* level was only increased in the PA‐treated group. I) mRNA levels of *HMGA1* in *HMGA1*‐KO Hep3B cells. J) Western blots of HMGA1 in *HMGA1*‐KO Hep3B cells. K) Western blots of ISG15 in *HMGA1*‐WT and *HMGA1*‐KO cells treated with 100 µM of PA for 24 h. L) mRNA levels of *ISG15* in *HMGA1*‐WT and *HMGA1*‐KO cells treated with 100 µM of PA for 24 h. M) Overexpression of HMGA1 (plasmid transfection gradient: 0, 0.1, 0.2, 0.4, 0.8 µg) enhanced the luciferase activity driven by the *ISG15* promoter (2 kb upstream of *ISG15*) in Hep3B cells. N) ChIP analysis of HMGA1 occupancy on the different segments on *ISG15* promoter in Hep3B cells with or without 100 µM of PA treatment. O) Deletion of indicated *ISG15* promoter fragments attenuated the luciferase activity induced by HMGA1 in Hep3B cells. Compared with the WT group, in *ISG15* promoter constructs with these two sites deleted, HIGMA1‐induced promoter activity was significantly attenuated. Data shown as mean ± SD. *p < 0.05; **p < 0.01; ***p < 0.001.

To explore whether HFD‐induced ISG15 upregulation is through IRF signaling, the expression level of *Irf3* in mouse liver was investigated. The mRNA level of *Irf3* in HCC tissues was downregulated in HFD‐fed mice (**Figure** **2**A), with a trend toward decreased protein levels (Figure 2B,C), in agreement with previous reports that hepatic Irf3 is downregulated by HFD‐feeding.^[^ ^28^, ^29^, ^30^ ^]^ Moreover, we did not observe a significantly altered phosphorylated Irf3 level in HCC tissues between HFD‐ and NCD‐fed groups (Figure 2C), suggesting that Irf3 may not play a major role in upregulating hepatic Isg15 in HFD‐fed mice.

To identify transcription factors/cofactors that affect *Isg15* expression under HFD‐stress, transcription factor prediction tools JASPAR (http://jaspar.genereg.net), TFDB (http://bioinfo.life.hust.edu.cn/HumanTFDB), and hTFtarget (http://bioinfo.life.hust.edu.cn/hTFtarget) were used. In addition to *Irf3*, ten common candidates for transcription factors/cofactors that regulate *Isg15* expression were suggested (Figure 2D). We further compared the relative mRNA levels of these ten candidates in HCC tissues, between the NCD‐ and HFD‐fed groups from our previous RNA sequencing data,^[^ ^8^ ^]^ and *Hmga1* was the only differentially expressed gene (Table S5, Supporting Information). High mobility group A1 (HMGA1) is an essential nonhistone chromatin structural protein, participating in a myriad of cellular processes, including malignant transformation, embryogenesis and transcriptional regulation.^[^ ^31^ ^]^ In HCC tumors from HFD‐fed mice (DEN+HFD), *Hmga1* was upregulated at both mRNA and protein levels (Figure 2B,C,E). Moreover, overexpression of HMGA1 significantly upregulated ISG15 in Hep3B and HepG2 cells (Figure 2F), without affecting the levels of other ISGs such as interferon induced protein with tetratricopeptide repeats 1 (*IFIT1*) and radical S‐adenosyl methionine domain containing 2 (*RSAS2*) (Figure S1E,F, Supporting Information). Palmitic acid (PA) was used to determine whether high‐fat intake increases *ISG15* and *HMGA1* expression. PA treatment upregulated both ISG15 and HMGA1 levels in HCC cell lines (Figure 2G,H), but did not affect the nuclear localization of pIRF3 (Figure S1G, Supporting Information), suggesting that high fat stress induces HMGA1 without affecting IRF3 signaling.

To verify whether PA treatment upregulates ISG15 through HMGA1, we constructed a *HMGA1*‐KO Hep3B cell line (Figure 2I,J). PA‐induced ISG15 upregulation was significantly reduced in *HMGA1*‐KO cells (Figure 2K,L), suggesting that HMGA1 is required for PA‐induced ISG15 upregulation. Luciferase assays demonstrated that HMGA1 upregulated the promoter activity of *ISG15* (Figure 2M). Chromatin immunoprecipitation assay (ChIP) demonstrated that HMGA1 binds within the region between −1000 and −2000 bp upstream of the *ISG15* gene, and PA treatment further enhanced this binding (Figure 2N). To map the HMGA1 binding promoter region, the transcription factor prediction tool JASPAR was used, and two potential upstream sites within the −2000 and −1000 bp were predicted (−1112 to −1105 bp and −1251 to −1244 bp upstream of *ISG15* gene). Luciferase assays indicated that in *ISG15* promoter constructs with these two sites deleted, HIGMA1‐induced promoter activity was significantly attenuated (Figure 2O), suggesting these two *ISG15* promoter sites are critical for HMGA1‐induced transcriptional activity. Together, these results suggested ISG15 was transcriptionally upregulated, at least in part, by HMGA1, especially following fat stimulation.

HMGA1 was consistently upregulated in HCC samples (Figure S1H, Supporting Information, data from GEPIA); moreover, HMGA1 levels were further elevated in HCC tissues with steatosis compared with those from steatosis‐free HCC clinical samples (Figure S1I, Supporting Information; GEO GSE193084).^[^ ^32^ ^]^ Correlation analysis from cBioPortal indicated a positive correlation between the levels of HMGA1 and ISG15 (Figure S1J, Supporting Information), and significantly lower overall survival rates were found in HCC patients with high HMGA1 levels (Figure S1K, Supporting Information, data from UALCAN). Furthermore, similar to ISG15, HMGA1 was also upregulated in clinic NAFLD samples compared with healthy liver tissues (Figure S1L, Supporting Information, GEO GSE130970),^[^ ^27^ ^]^ suggesting a potential association between HMGA1 and fatty liver. On the other hand, the expression level of another architectural DNA and nucleosome‐binding protein member of the high mobility group protein (HMG) family, high‐mobility group box‐1 (HMGB1), was not positively associated with ISG15 in HCC patients from cBioPortal (Figure S1M, Supporting Information).

### 2.3 ISG15 Inhibits PA‐Induced Apoptosis of HCC Cells

**Figure 3:**
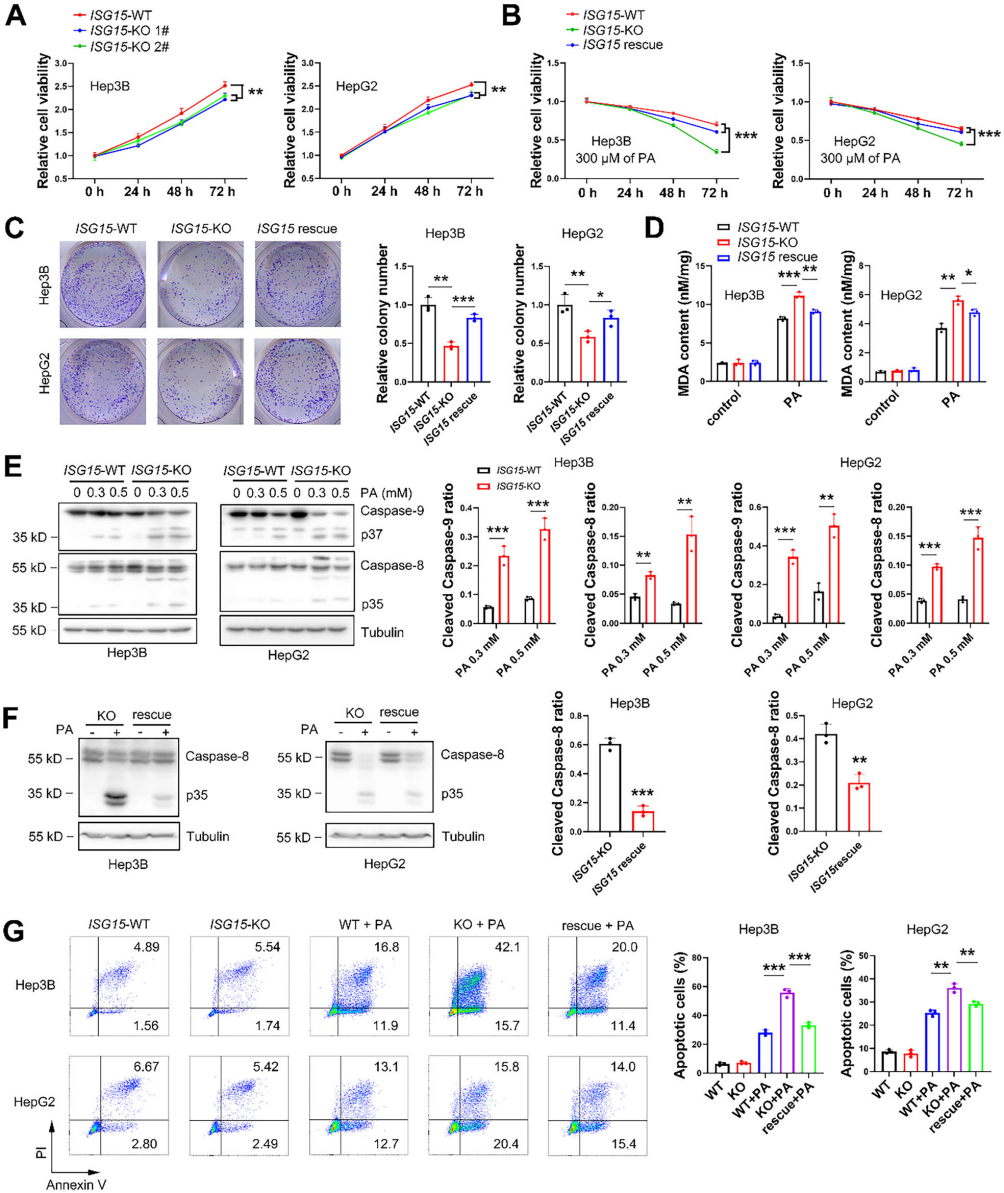
ISG15 abrogates fat stress induced cytotoxicity in HCC cells. A) Viability of *ISG15*‐WT/KO Hep3B and HepG2 cells in PA‐free condition determined by MTT analysis. B) Viability of *ISG15*‐WT/KO and ISG15‐rescued Hep3B and HepG2 cells treated with 300 µM of PA. C) Colony formation of *ISG15*‐WT/KO and ISG15‐rescued Hep3B and HepG2 cells under 300 µM of PA treatment for 72 h (left), and quantification results (right). D) MDA contents of *ISG15*‐WT/KO and ISG15‐rescued Hep3B and HepG2 cells with or without 300 µM of PA treatment. E) Western blots and the quantitative ratio of the cleaved to full‐length of Caspase‐9/Caspase‐8 in *ISG15*‐WT/KO Hep3B and HepG2 cells under 300 µM of PA treatment for 48 h. F) Western blots and the quantitative ratio of the cleaved to full‐length of Caspase‐8 in *ISG15*‐KO and ISG15‐rescued Hep3B and HepG2 cells under 300 µM of PA treatment for 48 h. G) Apoptosis of *ISG15*‐WT/KO and ISG15‐rescued Hep3B and HepG2 cells under 300 µM of PA treatment for 48 h determined by FACS analysis. Data shown as mean ± SD. *p < 0.05; **p < 0.01; ***p < 0.001.

A previous study reported that ISG15 could stimulate HCC cell growth.^[^ ^21^ ^]^ We consistently observed that deletion of ISG15 (Figure S2A, Supporting Information) resulted in reduced viability and migration (**Figures** **3**A, and S2B, Supporting Information); whereas ISG15 overexpression enhanced migration in Hep3B and HepG2 cells (Figure S2C, Supporting Information), suggesting that ISG15 promotes the proliferation of HCC cells.

To study the effects of fat accumulation on HCC cell growth, PA was administrated to Hep3B and HepG2 cells. At a low concentration of PA (25 µM), cell growth was promoted (Figure S2D, Supporting Information); whereas at higher concentrations (50–500 µM), cellular proliferation was inhibited (Figure S2D, Supporting Information). To simulate the cytotoxic high‐fat environment, 300 µM of PA was used, which induced obvious cytotoxicity in Hep3B and HepG2 cells (Figure S2D, Supporting Information) and caused even greater cytotoxicity in *ISG15*‐KO cells (Figure 3B), while restoration of ISG15 (*ISG15* rescue) significantly attenuated the cytotoxicity (Figure 3B). Also, *ISG15* deletion caused significantly decreased colony formation in Hep3B and HepG2 cells treated with 300 µM of PA, while restoration of ISG15 rescued the colony formation ability (Figure 3C).

We further analyzed malondialdehyde production (MDA, a lipid peroxidation product) and apoptosis to evaluate the inhibitory effect of ISG15 on high‐fat intake induced cytotoxicity. PA treatment triggered an increased level of MDA, and ISG15 deletion further elevated MDA, while restoration of ISG15 normalized the MDA level (Figure 3D). These results suggested that ISG15 may reduce PA‐induced lipid peroxidation in HCC cells. Caspase‐9 plays a critical role in mitochondria‐mediated apoptosis induced by oxidative damage,^[^ ^33^ ^]^ Western blots demonstrated enhanced PA‐induced cleavage of Caspase‐8 as well as Caspase‐9 in *ISG15*‐KO cells (Figure 3E), whereas restoration of ISG15 attenuated Caspase‐8 cleavage (Figure 3F), suggesting an anti‐apoptotic role of ISG15 in PA‐induced apoptosis. To verify the anti‐apoptotic role of ISG15, an Annexin V‐PI dual staining assay using a fluorescence activating cell sorter (FACS) was employed. PA treatment triggered massive apoptosis in wildtype cells, which was further enhanced in *ISG15*‐KO cells and was significantly rescued when ISG15 was reconstituted (Figure 3G). These results suggest that ISG15 may inhibit PA‐induced apoptosis in HCC cells.

### 2.4 ISG15 Reduces ROS and Promotes Glutathione Production

**Figure 4:**
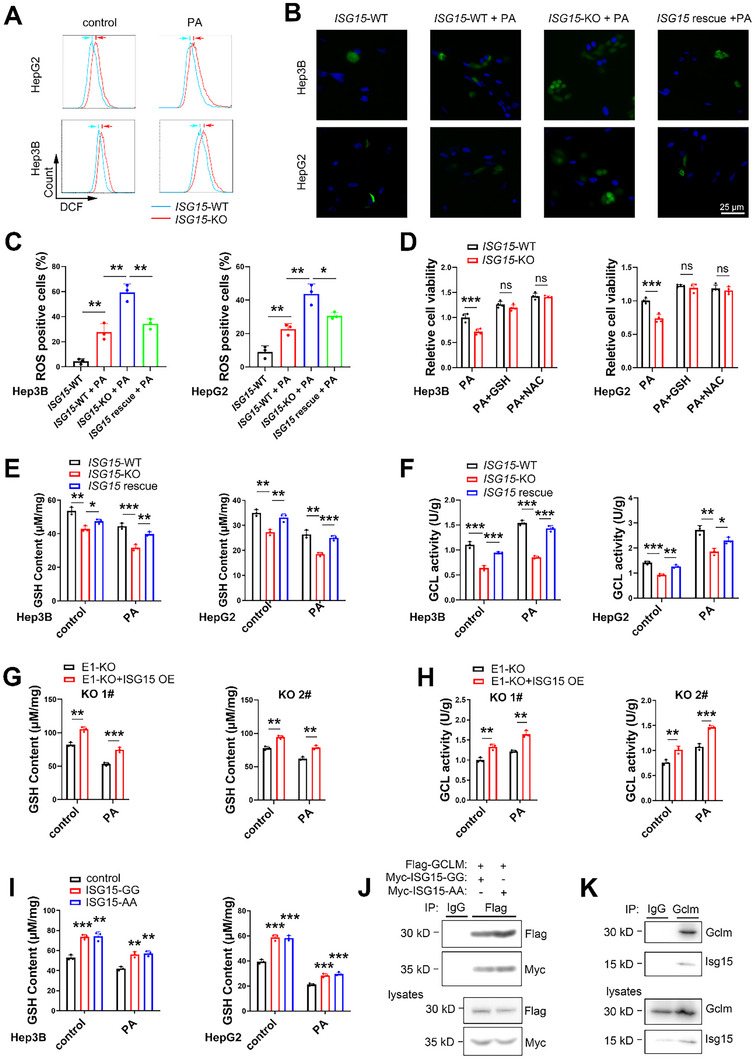
ISG15 inhibits apoptosis of HCC cells by increasing glutathione and reducing ROS. A) ROS levels of *ISG15*‐WT/KO Hep3B and HepG2 cells under 300 µM of PA treatments for 24 h determined by FACS analysis. B) Representative DCFH‐DA staining (Green fluorescence) images for ROS of *ISG15*‐WT/KO and ISG15‐rescued Hep3B cells treated with PA for 24 h. Cell nuclei were stained with Hoechst (blue fluorescence). C) Quantification results of ROS in Hep3B (left) and HepG2 (right) cells. D) Viability by MTT analysis of *ISG15*‐WT/KO Hep3B (left) and HepG2 (right) cells treated with PA (300 µM), or pre‐treated with glutathione (10 mM) or NAC (10 mM) for 12 h before PA treatment. (E) Total glutathione levels in *ISG15*‐WT/KO and ISG15‐rescued Hep3B (left) and HepG2 (right) cells treated with 300 µM of PA for 24 h. F) γ‐glutamate cysteine ligase activities in *ISG15*‐WT/KO and ISG15‐rescued Hep3B (left) and HepG2 (right) cells under 300 µM of PA treatment for 24 h. G) Total glutathione levels in two ISG15 overexpressing *UBA7*‐KO Hep3B cell lines (KO 1# and KO 2#) under PA treatments for 24 h. H) γ‐glutamate cysteine ligase activities in two ISG15 overexpressing *UBA7*‐KO Hep3B cell lines (KO 1# and KO 2#) under PA treatments for 24 h. I) Total glutathione levels in Hep3B and HepG2 cells overexpressing ISG15 mutants under 300 µM of PA treatments for 24 h. Compared with the control group, overexpressing ISG15 mutants increased total cellular glutathione levels with or without PA treatments. J) Co‐immunoprecipitation of GCLM and ISG15. HEK293T cells were co‐transfected with Flag‐GCLM and/or Myc‐ISG15‐GG/AA as indicated, anti‐Flag immunoprecipitates (top) and total lysates (bottom) were subjected to immunoblot with anti‐Flag antibody to reveal foreign GCLM, and anti‐Myc antibody to reveal foreign ISG15. K) Co‐immunoprecipitation of endogenous Gclm and Isg15 in mouse HCC tissues. Anti‐Gclm immunoprecipitate and total lysate of mouse tissues were subjected to immunoblot with anti‐Gclm and anti‐Isg15 antibodies. Data shown as mean ± SD. *p < 0.05; **p < 0.01; ***p < 0.001.

Fat accumulation causes elevation of ROS in liver cells, which induces oxidative stress and cytotoxicity that may lead to apoptosis suppressing cell proliferation.^[^ ^34^ ^]^ We tested whether ISG15 regulates cellular ROS level. Higher ROS levels were found in *ISG15*‐KO cells compared to the wildtype control cells, in the presence or absence of PA (**Figure** **4**A), while restoration of ISG15 reduced ROS level in *ISG15*‐KO cells (Figure 4B,C). We further tested whether ISG15 protected hepatic cells though ROS scavenging. Pretreatment with glutathione and N‐acetyl‐cysteine (NAC), rescued the enhanced cytotoxicity in *ISG15*‐KO cells (Figure 4D), suggesting that ISG15 may inhibit fat stress induced damage through reducing ROS.

Glutathione is the key endogenous ROS scavenger against oxidative stress,^[^ ^35^ ^]^ its level was significantly reduced in *ISG15*‐KO cells and was recovered by ISG15 restoration (Figure 4E). The enzyme activity of γ‐GCL was measured since it plays a critical role in glutathione generation (the first rate‐limiting enzyme of *de novo* synthesis of glutathione).^[^ ^15^ ^]^ γ‐GCL activity was reduced in *ISG15*‐KO cells, while ISG15 restoration significantly increased γ‐GCL activity (Figure 4F). To study whether ISG15 affects HCC cell proliferation by regulating γ‐GCL activity, a GCL inhibitor buthionine sulfoximine (BSO) was used,^[^ ^36^ ^]^ which significantly suppressed γ‐GCL activity (Figure S3A, Supporting Information). The ability of ISG15 to affect cell growth under PA stress was also attenuated upon BSO treatment (Figure S3B, Supporting Information), confirming that ISG15 affects HCC growth by enhancing γ‐GCL activity.

The γ‐GCL holoenzyme has a catalytic GCLC subunit, and a modifier GCLM subunit.^[^ ^15^ ^]^ Knockout or overexpression of ISG15 did not affect the expression levels of GCLM or GCLC (Figure S3C,D, Supporting Information), suggesting that ISG15 regulates γ‐GCL activity without affecting γ‐GCL expression. Next, we investigated whether ISG15 regulates glutathione levels through covalent ISGylation of GCLM or GCLC. The ISGylation specific E1 activating enzyme ubiquitin like modifier activating enzyme 7 (UBA7) was knocked out in Hep3B and HepG2 cells, which nearly abolished ISGylation (Figure S3E, Supporting Information), but similar glutathione levels were observed in *UBA7*‐KO and the control WT cells (Figure S3F, Supporting Information). Moreover, overexpression of ISG15 still increased γ‐GCL activity and glutathione levels in *UBA7*‐KO Hep3B (Figure 4G,H) and HepG2 cells (Figure S3G,H), suggesting that ISGylation is not required for the enhanced glutathione synthesis. Overexpression of ISG15‐AA, an ISG15 mutant with the C‐terminal di‐glycine (GG) substituted by di‐alanine (AA) that disrupts covalent ISGylation (Figure S3I, Supporting Information), consistently increased glutathione levels in both Hep3B and HepG2 cells (Figure 4I). These results suggest that ISG15 regulates glutathione production independent of ISGylation.

Since ISG15 enhanced γ‐GCL enzymatic activity without affecting its expression level or through ISGylation (Figure S3C,D, Supporting Information, Figure 4G–I), we next investigated whether ISG15 interacts with GCLM and/or GCLC subunits by using CoIP assays. While no direct interaction between GCLC and ISG15 was detected (Figure S3J, Supporting Information), both free forms and conjugated ISG15 were detected in the CoIP of GCLM (Figure 4J,K and Figure S3K, Supporting Information), suggesting that GCLM can be simultaneously ISGylated and noncovalently bound by free ISG15.

### 2.5 ISG15 Promotes GCLM/GCLC Interaction

**Figure 5:**
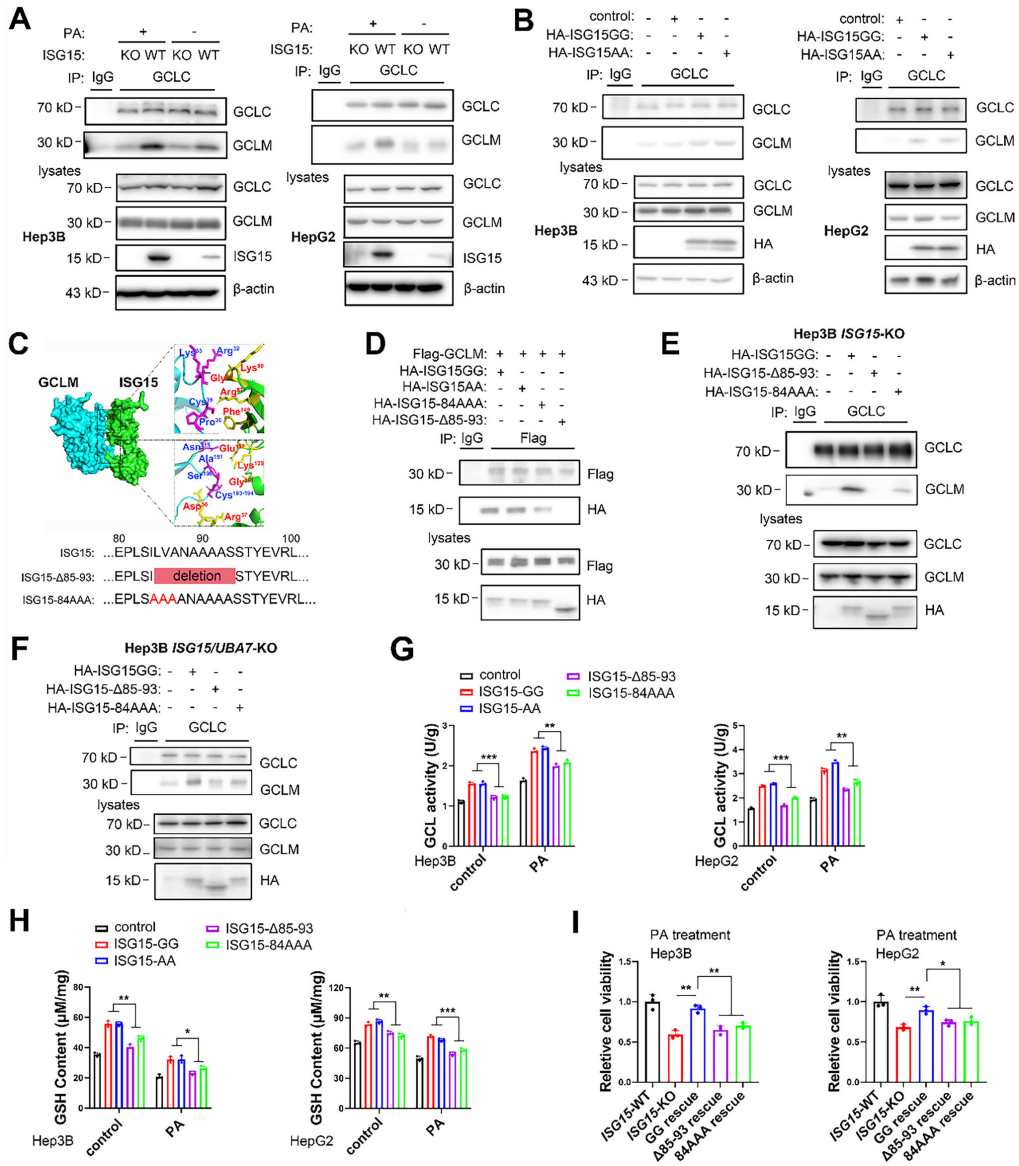
ISG15 upregulates glutathione production by promoting the GCLC‐GCLM interaction. A) Co‐immunoprecipitation of GCLM/GCLC and ISG15. *ISG15*‐WT/KO Hep3B (left) and HepG2 (right) cells were treated with PA as indicated, anti‐GCLC immune‐precipitates (top) and total lysates (bottom) were subjected to immunoblot with GCLC, GCLM and ISG15 antibodies. 100 µM of PA was added 24 h before cell lysis. B) *ISG15*‐KO Hep3B (left) and HepG2 (right) cells were co‐transfected with HA‐ISG15‐GG/AA as indicated. Anti‐GCLC immunoprecipitates (top) and total lysates (bottom) were subjected to immunoblot with GCLC, GCLM, and ISG15 antibodies. C) Representative structures of ISG15 (green) and GCLM (blue), and residues on the interaction interface are as labeled; and the primary sequences of ISG15‐Δ85‐93 and ISG15‐84AAA. D) Co‐immunoprecipitation of GCLM and indicated ISG15 constructs. HEK293T cells were co‐transfected with Flag‐GCLM and HA‐ISG15 mutants as indicated. Anti‐Flag immunoprecipitates (top) and total lysates (bottom) were subjected to immunoblot with anti‐Flag and anti‐HA antibodies. E,F) Co‐immunoprecipitation of GCLC/GCLM and indicated ISG15 constructs in *ISG15*‐KO (E) and dual knockout of *ISG15* and *UBA7* Hep3B cells (F). Cells were co‐transfected with HA‐ISG15 mutants as indicated. Anti‐GCLC immunoprecipitates (top) and total lysates (bottom) were subjected to immunoblot with anti‐GCLM and anti‐GCLC antibodies. G) γ‐glutamate cysteine ligase activities in *ISG15*‐KO Hep3B (left) and HepG2 (right) cells expressing HA‐ISG15 mutants with or without 300 µM of PA treatment. H) Total glutathione levels in *ISG15*‐KO Hep3B (left) and HepG2 (right) cells expressing HA‐ISG15 mutants with or without 300 µM of PA treatment. I) Viability of *ISG15*‐WT/KO Hep3B (left) and HepG2 (right) cells overexpressing HA‐ISG15 mutants with 300 µM of PA treatment for 48 h determined by MTT assay. Data shown as mean ± SD. *p < 0.05, **p < 0.01, ***p < 0.001.

We next investigated whether ISG15 regulates glutathione level through its interaction with GCLM. In Hep3B cells, knock‐down of GCLM using short hairpin RNA (shRNA) inhibited exogenous ISG15‐induced upregulated total glutathione (Figure S4A–C, Supporting Information), suggesting that endogenous GCLM is required for ISG15‐induced glutathione upregulation. Because heterodimerization of GCLM with the catalytic subunit GCLC forming the γ‐GCL holoenzyme significantly increases the enzyme activity,^[^ ^37^ ^]^ and ISG15 interacts with GCLM (Figure 4J,K), we speculated that ISG15 may affect γ‐GCL activity by facilitating the binding of GCLM and GCLC. To test this hypothesis, we performed a set of CoIP assays and found that compared to *ISG15*‐WT cells, the interaction of endogenous GCLM/GCLC was reduced in *ISG15*‐KO Hep3B and HepG2 cells (**Figure** **5**A); while overexpression of wildtype ISG15 or ISG15‐AA (ISGylation‐free mutant) all enhanced the GCLM‐GCLC interaction (Figure 5B). These data suggested that ISG15 enhances GCLM‐GCLC interaction in an ISGylation independent manner.

To determine whether the noncovalent binding between ISG15 and GCLM promotes the GCLM‐GCLC interaction, we performed in silico molecular simulation using AlphaFold that enables precise modeling of protein‐protein interactions (PPIs).^[^ ^38^ ^]^ The simulation of the non‐conjugated ISG15/GCLM complex suggested four potential binding regions on ISG15 (1‐157), spanning residues 54–58, 85–93, 125–130, and 141–151, respectively (Figure S4D and Table S6, Supporting Information). Accordingly, two ISG15 truncation constructs (ISG15‐1‐80 and ISG15‐1‐120, Figure S4D, Supporting Information) were generated and CoIP experiments with GCLM were performed. The CoIP results demonstrated that construct 1–120 interacted with GCLM whereas construct 1–80 did not (Figure S4E, Supporting Information), suggesting that the region 85–93 of ISG15 is critical for binding with GCLM (Figure S4D, Supporting Information). Accordingly, we constructed two additional ISG15 mutants (Figure 5C), ISG15‐84AAA (which mutate I^84^L^85^V^86^ to A^84^A^85^A^86^ to disrupt the potential hydrophobic pocket) and ISG15‐Δ85‐93 (85‐93 deletion), which showed similar expression level and ISGylation ability as that of wildtype ISG15 (ISG15‐GG) (Figure S4F, Supporting Information). CoIP assays demonstrated that compared with wildtype ISG15, ISG15‐84AAA and ISG15‐Δ85‐93 showed an obviously attenuated interaction with GCLM (Figure 5D). Further CoIP assays using *ISG15*‐KO cells indicated that, ISG15‐84AAA and ISG15‐Δ85‐93 had attenuated GCLM‐GCLC interaction compared with ISG15‐GG (Figure 5E), suggesting that the interaction between ISG15 and GCLM is critical for the GCLM‐GCLC interaction. To exclude the possibility of interference by endogenous ISG15 and ISGylation, we constructed a Hep3B cell line with knock out of both *ISG15* and *UBA7* (*ISG15/UBA7* dual‐KO) (Figure S4G, Supporting Information). CoIP assays in *ISG15/UBA7* dual‐KO cells also demonstrated that ISG15‐84AAA and ISG15‐Δ85‐93 displayed decreased enhancement of the GCLM‐GCLC interaction compared with that of ISG15‐GG (Figure 5F). Moreover, compared with wildtype ISG15, both mutants demonstrated impaired ability to enhance γ‐GCL activity and glutathione synthesis (Figure 5G,H), as well as attenuating the rescue of PA‐induced inhibition of viability in Hep3B and HepG2 cells (Figure 5I). Together, these results suggest that ISG15 non‐covalently binds GCLM to enhance the GCLM‐GCLC interaction and promote glutathione synthesis.

In silico simulations suggested that ISG15 promoted the interaction between GCLM and GCLC, as indicated by the distinct decrease in Δ^i^G of the GCLM/GCLC heterodimer (Table S7 and Figure S4H, Supporting Information), which suggested that ISG15, GCLM and GCLC may form a complex in the cell. To test this hypothesis, GCLM and GCLC were overexpressed in Hep3B cells in the presence or absence of exogenous ISG15 (Figure S4I, Supporting Information), and CoIPs were performed. Exogenous ISG15 enhanced the GCLM‐GCLC interaction, and notably, in cells overexpressing ISG15/GCLM/GCLC, co‐immunoprecipitation of ISG15‐GCLC was detected (Figure S4I, Supporting Information). Since direct interaction between ISG15‐GCLC was hardly detected in the absence of foreign GCLM (Figure S3J, Supporting Information), these results suggest that ISG15 may bind to GCLM to form an ISG15/GCLM/GCLC complex. Moreover, according to biased residues close to the potential ISGylation sites,^[^ ^39^ ^]^ out of 17 lysine residues in GCLM, 4 potential ISGylation sites (K49, K80, K158 and K169) were predicted (Table S8, Supporting Information), and none of them were located at the non‐covalent interaction interfaces of the GCLM/GCLC or GCLM/ISG15 complexes (Figure S4J, Table S6, Supporting Information).

### 2.6 ISG15 is Critical for High Fat Diet‐Induced HCC Progression

To investigate the role of ISG15 in dietary obesity, *Isg15*‐KO (*Isg15* ^‐/‐^) and WT mice (*Isg15* ^+/+^) were fed a NCD or HFD for 28 weeks (Figures S5A,B, Supporting Information). As expected, HFD feeding significantly increased body and liver weight, as well as fatty liver (Figure S5C–E, Supporting Information). Under NCD or HFD feeding, the phenotype of *Isg15* ^‐/‐^ mice was similar to that of WT mice, including embryonic development (the birth rate of *Isg15* ^‐/‐^ mice followed Mendelian predictions), body and liver weight, and liver function (Figure S5C–E, Supporting Information). Also, *Isg15*‐KO did not affect hepatic Gclm/Gclc protein levels (Figure S5B, Supporting Information) while showed elevated ROS levels (Figure S5F, Supporting Information).

**Figure 6:**
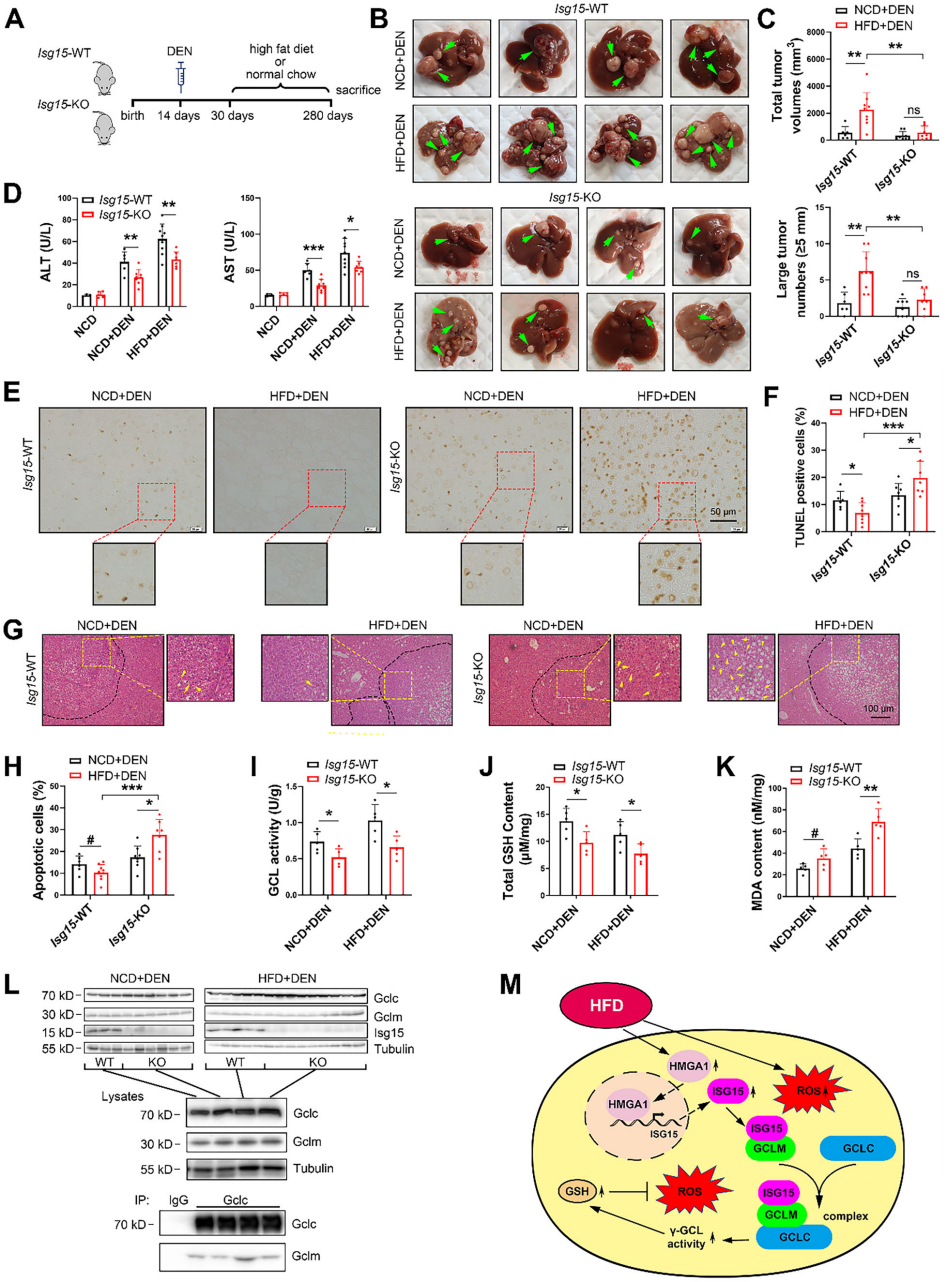
Ablation of *Isg15* inhibits HCC progression in HFD‐fed mice. A) Experiment flowchart of DEN‐induced HCC in HFD‐fed mice. B) Representative liver images for each group. Arrows indicate tumors. C) Quantification results of total tumor volumes and number of large tumors. D) Serum AST and ALT levels of mice from indicated groups. E,F) Representative images of TUNEL staining (E) and quantification results (F) in mice HCC samples. G) Representative H&E staining in mice HCC samples. Dotted lines indicate tumor outlines; arrows indicate Councilman bodies (apoptotic hepatic cells). H) Quantification results of apoptotic cells in mice HCC samples. I) γ‐glutamate cysteine ligase activities in mice HCC samples. J) Total glutathione levels in mice HCC samples. K) MDA contents in mice HCC samples. L) Co‐immunoprecipitation of Gclc/Gclm in mouse HCC samples. HCC samples were subjected to immunoblot with anti‐Gclm, anti‐Gclc and anti‐Isg15 antibodies. Equal amounts of each protein sample from the same group were normalized and combined into 4 groups, and anti‐Gclc immunoprecipitates and total lysates were subjected to immunoblot with anti‐Gclm and anti‐Gclc antibodies. M) Schematic model of pathways leading to development of HCC. High‐fat intake contributes to HCC development; however, fat accumulation also brings about cytotoxic effects such as excessive ROS, which leads to apoptosis and prevents HCC progression. HCC cells under high‐fat environment constrain harmful ROS by enhancing glutathione synthesis though upregulating ISG15. In this process, upregulated HMGA1 enhances ISG15 transcription, upregulated ISG15 subsequently noncovalently binds with GCLM and increases the GCLM‐GCLC interaction to form an ISG15/GCLM/GCLC complex, which has increased γ‐GCL enzymatic activity that promotes glutathione synthesis to protect HCC cells from ROS‐induced apoptosis. Data shown as mean ± SD. *p < 0.05; **p < 0.01; ***p < 0.001; # 0.05 < *p* < 0.07.

In DEN‐induced HCC models (**Figure** **6**A), while HFD feeding significantly increased the total volume and number of large HCC tumors (diameters ≥ 5 mm) in WT mice, HCC progression was significantly inhibited in HFD‐fed *Isg15‐*KO mice (Figure 6B,C, Supporting Information). The levels of serum glutamic oxaloacetic transaminase (AST) and glutamic pyruvic transaminase (ALT) were significantly reduced in *Isg15‐*KO mice compared to WT (Figure 6D) which suggested improved liver function by *Isg15* deletion. Terminal deoxynucleotidyltransferase‐mediated dUTP‐biotin nick end labeling (TUNEL) and hematoxylin‐eosin staining (H&E) staining demonstrated an increased proportion of apoptotic cells in tumor tissues from *Isg15* KO mice (Figure 6E–H). In addition, immunohistochemistry (IHC) staining of platelet endothelial cell adhesion molecule‐1 (PECAM‐1/CD31) and marker of proliferation Ki‐67 (Ki67) suggested that *Isg*15 KO resulted in attenuated angiogenesis and proliferation in HCC tissues (Figure S5G–J, Supporting Information). Intriguingly, the HFD has been reported to reduce apoptosis of HCC cells.^[^ ^5^ ^]^ Similarly, we found that the HFD suppressed apoptosis of HCC cells in *Isg15*‐WT mice (Figure 6E,F). In contrast, *Isg15*‐KO mice on a HFD showed enhanced apoptosis in HCC cells compared with those from NCD‐fed mice (Figure 6E–H), suggesting an important anti‐apoptotic role of ISG15 in HCC cells under HFD challenge. Moreover, compared to samples from WT mice, HCC tissues from *Isg15*‐KO mice showed reduced γ‐GCL enzymatic activity (Figure 6I), and decreased glutathione levels (Figure 6J). HCC tissues from *Isg15*‐KO mice also displayed significantly increased MDA levels compared to the *Isg15*‐WT group (Figure 6K), suggesting Isg15 may reduce HFD‐induced lipid peroxidation in vivo. Importantly, *Isg15*‐deletion did not alter endogenous Gclm/Gclc levels but attenuated the interaction between Gclm and Gclc in mice HCC tissues (Figure 6L). Together, the animal study suggests that ISG15 plays a key role in HFD‐induced HCC progression, whereas *Isg15*‐deletion induces apoptosis and suppresses HFD‐promoted HCC progression.

## 3 Discussion

ISG15 can be induced by IFN‐α/β through the Janus kinase (JAK) and signal transducer and activator of transcription (STAT) pathway.^[^ ^16^ ^]^ In addition, IFN‐γ, retinoic acid, lipopolysaccharide and DNA damage also trigger ISG15 expression through IRF3 signaling.^[^ ^22^ ^]^ We showed in this study that 36 weeks of HFD‐feeding upregulated Isg15 without significantly elevating Irf3 phosphorylation (Figure 2B), suggesting there may be alternative pathways that regulate ISG15. Our data demonstrate that hepatic HMGA1 is upregulated upon HFD stress (Figure 2B,E), and PA induces ISG15 expression in HCC cells through an HMGA1‐dependent mechanism (Figure 2K). Moreover, in addition to tumor tissues, *HMGA1* and *ISG15* were also upregulated in hepatic and renal steatosis (Figure S1C,D,L, Supporting Information), suggesting a correlation between fat accumulation and HMGA1‐ISG15 levels may exist in multiple tissues and diseases. We also showed that HMGA1 binds within a region −1000 to −2000 bp upstream of *ISG15* and promotes *ISG15* transcription, especially under high‐fat stress (Figure 2N). Previous studies reported that high HMGA1 levels predict a poor prognosis in diverse tumors, such as breast, liver, lung, ovarian, cervical and colorectal cancers.^[^ ^40^ ^]^ As a chromatin‐associated protein, HMGA1 is involved in transcription regulation by two mechanisms. One involves global activation of gene expression, and the other relates to its transcription cofactor function that regulates specific target genes like *IFN‐β*.^[^ ^31^ ^]^ The mechanism by which HMGA1 upregulates ISG15 is worthy of further study.

As genes downstream of NRF2 signaling, the γ‐GCL subunits GCLC and GCLM are often upregulated in solid tumors which predicts poor prognosis.^[^ ^41^, ^42^ ^]^ Increased GCLM and GCLC levels could enhance glutathione concentration, which blunts the impact of ROS and confers resistant to chemotherapy treatment in cancer cells.^[^ ^43^ ^]^ However, the NRF2 pathway is often inactivated or inhibited in liver diseases such as NAFLD,^[^ ^11^, ^12^ ^]^ and little is known about how HCC cells control glutathione synthesis during the NAFLD to HCC progression. Here, we demonstrate a new γ‐GCL activity regulation pathway, which is independent of γ‐GCL expression levels and may play a role in HFD‐promoted HCC development. ISG15 interacts with GCLM but not GCLC (Figure S3J, Supporting Information) to form an ISG15/GCLM/GCLC complex (Figure S4I, Supporting Information), and non‐covalently GCLM‐bound ISG15 stabilizes the GCLM/GCLC complex (Figure S4H, Supporting Information). Together, under high‐fat stress, upregulated ISG15 non‐covalently binds GCLM to form a ISG15/GCLM/GCLC complex with enhanced γ‐GCL activity, thus increasing glutathione production and playing an oncogenic role in HCC cells by reducing ROS‐induced apoptosis (Figure 6M), which may be further explored to develop γ‐GCL inhibitors for the treatment of liver and lung cancers by promoting apoptosis. Moreover, as a risk factor, dietary obesity plays important roles in the development and progression of many solid tumors, such as colorectal, breast, pancreatic and gastric cancers.^[^ ^44^ ^]^ For example, HFD‐feeding enhances adipocytes’ secretion of microsomal triglyceride transfer protein, which induces glutathione synthesis and reduces ROS generation, and eventually promotes colorectal cancer.^[^ ^45^ ^]^ Considering that ISG15 is upregulated in most types of cancers,^[^ ^22^ ^]^ the ISG15‐γ‐GCL‐glutothione axis may function broadly in cancer development in addition to HCC, which warrants future study.

Increasing evidence suggests that ISG15 plays a critical role in cancer development and progression. Through covalent ISGylation, ISG15 regulates oncogenic pathways, such as kirsten rat sarcoma 2 viral oncogene homolog (KRAS) and mitogen‐activated protein kinases (MAPK) pathways, by affecting the stability and/or function of the substrate proteins.^[^ ^22^ ^]^ Additionally, extracellular free ISG15 can function as an immunomodulatory cytokine, regulating immune cell function against cancer.^[^ ^22^ ^]^ However, little is known about the role of intracellular unconjugated free ISG15. Our results revealed a new function for nonconjugated ISG15 in regulating glutathione synthesis in HCC cells. Although glutathione plays a vital anti‐apoptotic role for cancer cells to adapt to high‐ROS conditions, regulation of its synthesis is not fully understood. For example, without activation of the ROS and glutathione regulator NRF2 signaling, the oxidative stress levels in steatosis associated chronic liver disease (CLD) were similar to those from non‐steatosis CLD samples;^[^ ^46^ ^]^ moreover, considering the unsatisfactory progress of clinical trials targeting NRF2,^[^ ^47^ ^]^ it is likely that there are additional anti‐oxidation pathways in cancer cells. Our finding that the ISG15‐γ‐GCL‐glutothione axis may prevent ROS‐induced apoptosis in HCC cells thus offers a new target for therapy against NRF2 inhibition insensitive cancers like HCC.^[^ ^47^ ^]^

## 4 Experimental Section

### Clinical Databases of Cancer

The relative gene expression levels in HCC samples were obtained from GEPIA (http://gepia.cancer‐pku.cn/). The survival rate analysis of HCC patients was obtained from UALCAN (http://ualcan.path.uab.edu/). Correlation analysis of gene expression in HCC samples was obtained from cBioPortal (http://www.cbioportal.org/). RNA sequencing data was obtained from NCBI (https://www.ncbi.nlm.nih.gov/).

### Clinical Samples and Approval

Liver paraffin sections from 20 patients with HCC were obtained from the Affiliated Hubei Cancer Hospital of Tongji Medical College. Collection and use of patient samples and patient characteristics were approved by the institutional review boards of the hospital (LHBCH2022ΥN‐015), all subjects included in the study provided informed consent. All research was conducted in accordance with both the Declarations of Helsinki and Istanbul.

### Mice, Diets, and Experimental Design

Mice were handled according to the Guidelines of the China Animal Welfare Legislation, as approved by the Committee on Ethics in the Care and Use of Laboratory Animals of College of Life Sciences, Wuhan University (WDSKY0201705‐2). C57BL/6 breeding pairs were from Hubei Center for Disease Control and Prevention. Systemic *Isg15*‐KO mice (*Isg15 ^–/–^*) were generated by Cyagen Biotech. (Suzhou, China). To generate HCC, male WT or *Isg15‐KO* mice were injected intraperitoneally with diethylnitrosamine (DEN) (25 mg k^−1^g body weight; Sigma, St. Louis, MO) 14 days after birth, then were fed with normal chow (NCD) or high fat diet (HFD) (60% kcal fat; Research Diets, New Brunswick, NJ) as previously reported.^[^ ^48^, ^49^ ^]^ All HCC bearing mice were sacrificed at 40 weeks of age. Total tumor volumes were determined by counting the tumors with diameter > 3 mm. Tumors with diameters ≥ 5 mm are designated as large tumors.

### Cell Culture

Human HCC cell lines Hep3B (China Center for Type Culture Collection, CCTCC, Wuhan, China) and HepG2 (Procell Biotech, Wuhan, China), and HEK293T (Procell Biotech) were cultured in DMEM media (Hyclone, Logan, UT) containing FBS. All cell lines were authenticated by short tandem repeat profiling and tested for mycoplasma contamination.

### Biochemical Measurements

Serum levels of alanine aminotransferase (ALT) and aspartate aminotransferase (AST) were measured using ALT/AST kits (Jiancheng, Nanjing, China) according to the manufacture's protocols.

### Histological, Immunohistochemical and TUNEL Staining

Clinical and mouse liver samples were embedded in paraffin and sectioned. For immunohistochemical staining, sections were incubated overnight with primary antibodies (Table S1, Supporting Information) and visualized by DAB substrate (Vector laboratories, Newark, CA). TdT‐mediated dUTP Nick‐End Labeling (TUNEL) assay was performed using a TUNEL Assay Kit (Beyotime, Shanghai, China). For histological evaluation, H&E staining were performed, and analyzed using Histo‐score by two individuals in a double‐blind procedure as reported.^[^ ^50^, ^51^ ^]^

### Knockout and Knockdown Cell Lines

CRISPR‐Cas9 based genome engineering was used as previously reported.^[^ ^52^ ^]^ Target sequences of gRNA for CRISPR‐Cas9 and small hairpin RNA (shRNA) are provided in Table S2, Supporting Information.

### qPCR, Western Blots and Luciferase Reporter Assays

qPCR and Western blots were performed as described.^[^ ^53^, ^54^ ^]^ Protein extraction from cytoplasm and nucleus was conducted using a protein extraction kit (Beyotime). Primers are provided in Table S3, Supporting Information, and antibodies for Western blots are provided in Table S1, Supporting Information. For reporter assay, a luciferase reporter containing an upstream 2 kb fragment of *ISG15* gene was constructed (Table S4, Supporting Information), and 12 h after transfection, luciferase activity was measured.

### Constructs and Transfections

Plasmids expressing Flag‐tagged GCLM, HMGA1; HA‐tagged ISG15‐GG, ISG15‐AA, ISG15‐1‐80, ISG15‐1‐120, ISG15‐Δ85‐93, ISG15‐84AAA, GCLC; as well as Myc‐tagged ISG15‐GG or ISG15‐AA, were constructed by standard procedures, using the eukaryotic expression plasmid pRK vector. Transfections were performed using Lipofectamine 2000 (Invitrogen, Carlsbad, CA).

### Chromatin Immunoprecipitation

ChIP was performed as described previously.^[^ ^55^ ^]^ The fold enrichment relative to input was measured by qPCR (primers provided in Table S4, Supporting Information).

### MTT, Colony Formation, and Migration Assay

Hep3B or HepG2 cells were transfected with the indicated plasmids. 12 h after transfection, the cells were cultured with or without palmitic acid (PA) of indicated concentrations. Methylthiazolyldiphenyl‐tetrazolium bromide (MTT), colony formation, and migration assays were performed as we previously reported.^[^ ^56^ ^]^

### Detection of γ‐GCL Activity, ROS, MDA, Annexin V and Glutathione

γ‐glutamate cysteine ligase (GCL) activity was detected using a γ‐GCL kit (Solarbio, Beijing, China). ROS content was measured using a ROS assay kit, MDA was detected using a lipid peroxidation MDA assay kit, glutathione level in tissue or cell lines was measured using a GSH and GSSG assay kit (all from Beyotime). Quantification of apoptotic cells was performed using an Annexin V apoptosis detection kit (Beyotime).

### Co‐Immunoprecipitation Assay

Cells were lysed with pre‐lysis buffer, and cell lysates were incubated with antibodies (Table S1, Supporting Information) or respective IgGs with Dynabeads Protein G (Thermo Scientific, Waltham, MA) overnight at 4 °C. After washing with pre‐lysis buffer containing 50 mM NaCl, beads were boiled in loading buffer and subjected to immunoblotting as previously reported.^[^ ^57^ ^]^

### Protein‐Protein Interaction Modeling

Protein‐protein interaction between ISG15, GCLM and GCLC were modeled by Alphafold2 repository.^[^ ^58^ ^]^ Query sequence and multiple sequence alignment (MSA) from MMseqs2 were input without templates. MSA generation and AlphaFold 2 predictions were performed via the ColabFold (https://colab.research.google.com/github/sokrypton/ColabFold). Modeling results were analyzed by PDBePISA (www.ebi.ac.uk/msd‐srv/prot_int/pistart.html). Binding sites of ISG15, GCLM and GCLC were analyzed by Δ^i^G (solvation energies) and RMSD. PPI‐contributing hot spots were selected and visualized by PyMOL 2.5.

### Statistical Analyses

Data were analyzed with GraphPad Prism. Statistical analysis was performed using two‐tailed Student's t‐test for two experimental groups, and one‐way ANOVA for multiple experimental groups without adjustment. Data are reported as the mean values with the standard deviation (SD). For cell experiments, at least three independent experiments were performed with similar results obtained. A *p*‐value of < 0.05 was considered statistically significant. For quantification of Western blot, ImageJ software was used to measure the relative intensity of each band, and the relative protein levels were normalized to levels of loading controls. The sample size (n) for each statistical analysis is presented in the figures.

## Conflict of Interest

The authors declare no conflict of interest.

## Author Contributions

Conceptualization: X.L., K.H., L.Z. Methodology: X.L., Y.X., Y.C., H.C., Y.Z., J.Y., Y.S., R.B.P., Y.L., A.P. Investigation: X.L., Q.M., Z.J., C.Z., L.S., C.M., Y.Z., C.W. Visualization: X.L. Supervision: K.H., L.Z. Writing: X.L., K.H., L.Z.

## Supporting information



Supporting Information

Supplemental Table 1

Supplemental Table 2

## Data Availability

The data that support the findings of this study are available from the corresponding author upon reasonable request.
