# Supplementary material for: ISG15 Enhances the Activity of γ‐Glutamate Cysteine Ligase to Suppress Apoptosis in High Fat Diet‐Promoted Hepatocellular Carcinoma
Source: Adv Sci (Weinh). 2025 Mar 24;12(19):2416401. doi: 10.1002/advs.202416401 (PMC12097128; doi:10.1002/advs.202416401)
Supplement: Supplementary file 1 — Supporting Information [file ADVS-12-2416401-s002.docx]

**Supporting Information**

***for***

**ISG15 Enhances the Activity of γ-Glutamate Cysteine Ligase to Suppress Apoptosis in High Fat Diet-Promoted Hepatocellular Carcinoma**

Xinran Liu^1^, Qiujin Ma^1^, Zhao Jia^1^, Yihao Zhou^2^, Churong Zou^1^, Yushuo Xiao^1^, Yuchen Chen^1^, Chuyao Ma^1^, Liangliang Song^1^, Jing Yang^1^, Chen Wang^1^, Huidie Xu^1^, Hong Chen^1^, Jiajian Shi^1^, Junqiu Yue^3^, Yu Sun^2^, Desheng Hu^4,5^, Robert B Petersen^6^, Yangkai Li^7^, Anlin Peng^8^,

Kun Huang^1^* & Ling Zheng^2^*

^1^ School of Pharmacy, Tongji Medical College and State Key Laboratory for Diagnosis and Treatment of Severe Zoonotic Infectious Diseases, Huazhong University of Science & Technology, Wuhan, China, 430030

^2^ College of Life Sciences, Wuhan University, Wuhan, China, 430072

^3^ Department of Pathology, Hubei Cancer Hospital, Tongji Medical College, Huazhong University of Science and Technology, Wuhan, China, 430079

^4^ Department of Integrated Traditional Chinese and Western Medicine, Union Hospital, Tongji Medical College, Huazhong University of Science and Technology, Wuhan, China, 430000

^5^ China-Russia Medical Research Center for Stress Immunology, Union Hospital, Tongji Medical College, Huazhong University of Science and Technology, Wuhan, China, 430000

^6^ Foundational Sciences, Central Michigan University College of Medicine, Mt. Pleasant, USA, MI 48859

^7^ Department of Thoracic Surgery, Tongji Hospital, Tongji Medical College, Huazhong University of Science and Technology, Wuhan, China, 430030

^8^ Department of Pharmacy, The Third Hospital of Wuhan, Tongren Hospital of Wuhan University, Wuhan, China, 430070

^*^**Corresponding authors**

Ling Zheng, Ph.D. Kun Huang, Ph.D.

College of Life Sciences Tongji School of Pharmacy

Wuhan University Huazhong University of Science & Technology

Wuhan, China, 430072 Wuhan, China, 430030

E-mail: [lzheng@whu.edu.cn](mailto:lzheng@whu.edu.cn) E-mail: [kunhuang@hust.edu.cn](mailto:kunhuang@hust.edu.cn)

**This supplementary file contains 5 supplementary figures and 8 supplementary tables.**


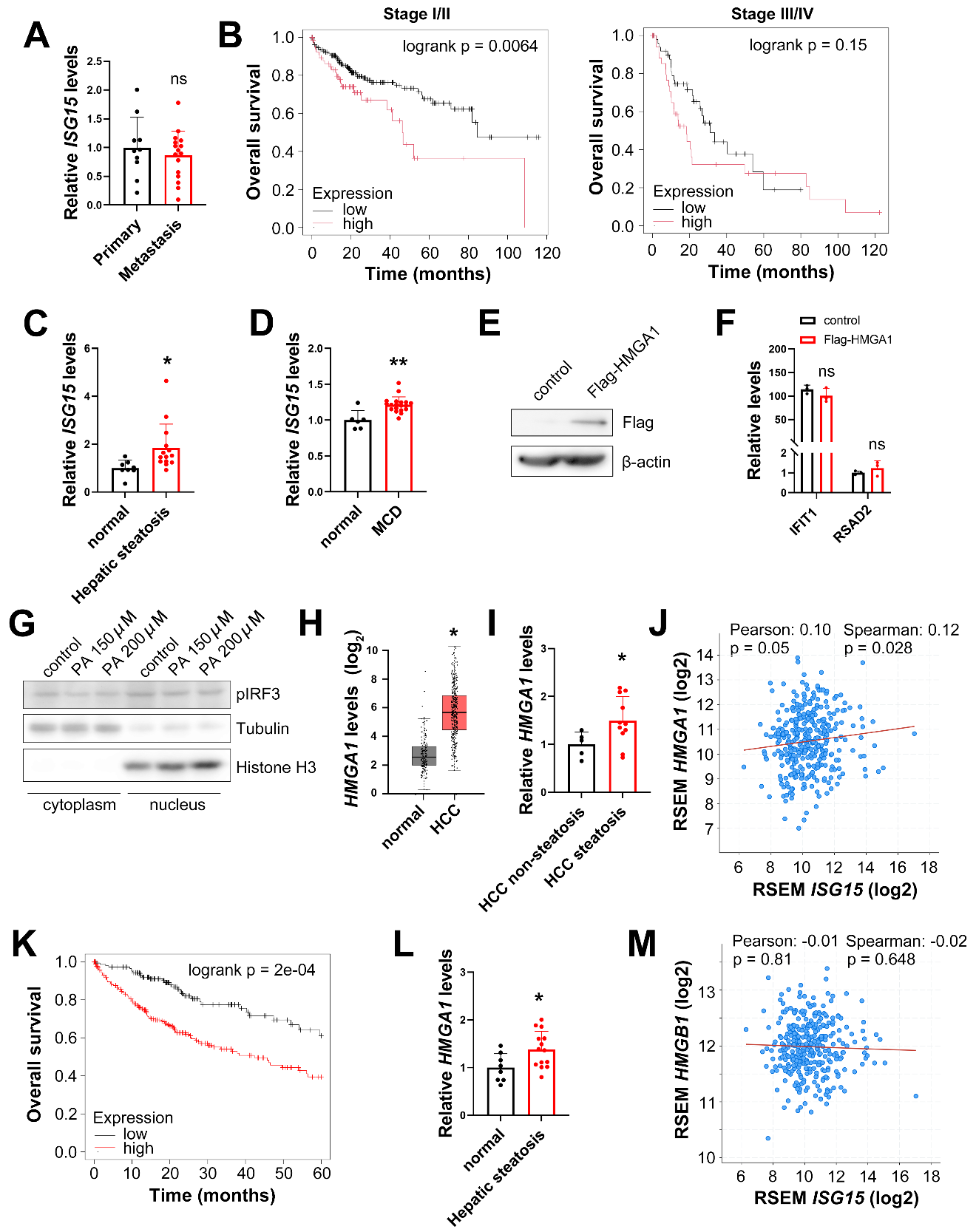


**Fig. S1. HMGA1 and ISG15 expression levels in clinical samples.** (**A**) The mRNA levels of *ISG15* in primary HCC and metastatic tumor samples. RNA-seq data was from GSE63018. (**B**) Over-all survival rates of HCC patients with high ISG15 expression levels (red) and low levels (black) in stage I/II (left) and stage III/IV (right) of HCC patients. Data from cBioPortal. (**C**) The mRNA levels of *ISG15* in NAFLD clinical samples. RNA-seq data from GEO (GSE130970). (**D**) The mRNA levels of *ISG15* in MCD clinical samples. RNA-seq data from GEO (GSE200828). (**E**) Western blots detecting Flag-HMGA1 in HepG2 cells after transfection of the plasmid expressing Flag-HMGA1. (**F**) The mRNA levels of *IFIT1* and RSAD2 in HepG2 cells after transfection of the plasmid expressing Flag-HMGA1. (**G**) Western blots detecting phosphorylated IRF3 in the cytoplasm and nucleus of HepG2 cells after PA treatment. (**H**) The mRNA levels of *HMGA1* in HCC and normal tissues. Data from GEPIA. (**I**) The mRNA levels of *HMGA1* in clinical HCC samples. RNA-seq data from GEO (GSE193084). (**J**) Correlation analysis of the expression levels of *HMGA1* and *ISG15* in clinical HCC samples. Data from cBioPortal. (**K**) Over-all survival rates of HCC patients with high HMGA1 expression levels (red) and low levels (black). Data from UALCAN. (**L**) The mRNA levels of *HMGA1* in NAFLD clinical samples. RNA-seq data from GEO (GSE130970). (**M**) Correlation analysis of the expression levels of *HMGB1* and *ISG15* in clinical HCC samples. Data from cBioPortal. Data shown as mean ± SD. ns, not significant; * p < 0.05; ** p < 0.01. *** p < 0.001.


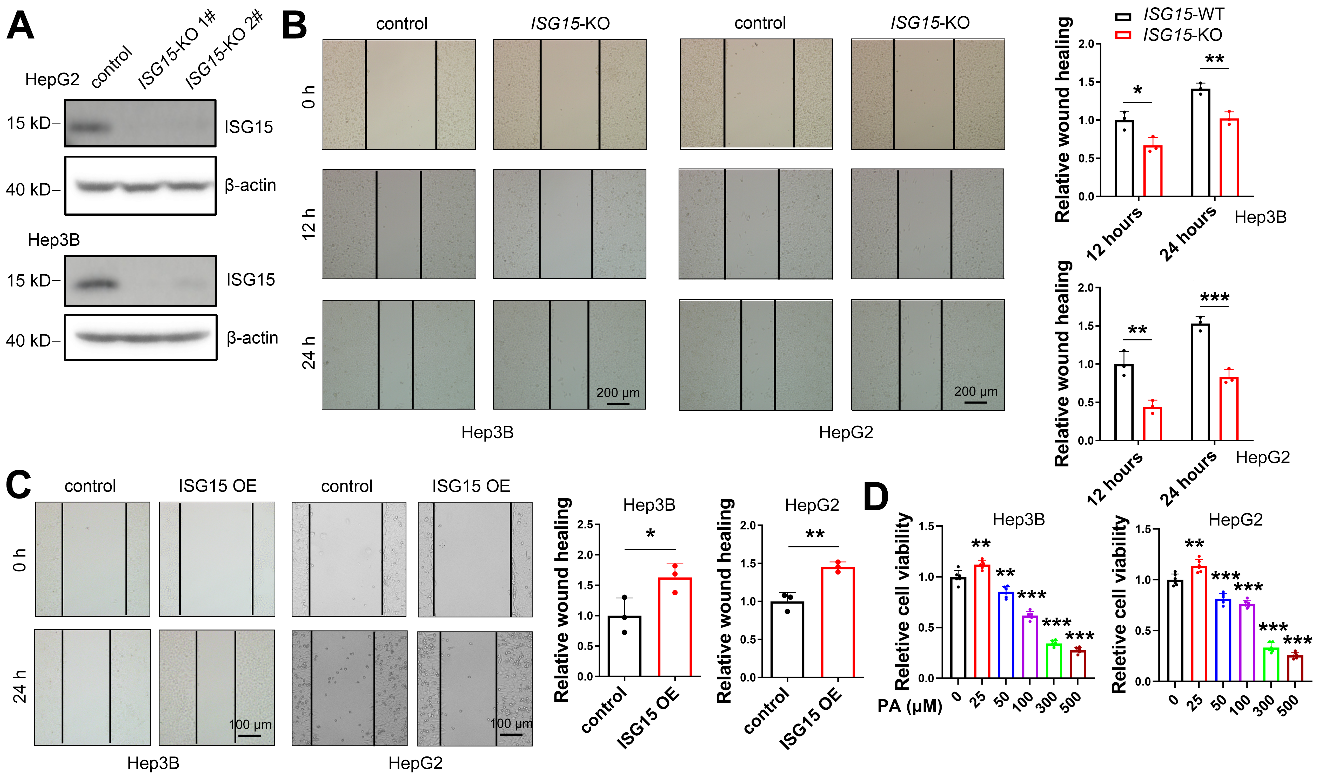


**Figure S2. ISG15 promotes HCC cell proliferation.** (**A**) Western blots of ISG15 in *ISG15*-KO Hep3B and HepG2 cells. (**B**) Migration of *ISG15*-WT/KO Hep3B and HepG2 cells in PA-free condition determined by wound healing assay. (**C**) Migration of ISG15-overexpressing (ISG15 OE) Hep3B and HepG2 cells in PA-free condition determined by wound healing assay. (**D**) Viability of Hep3B (left) and HepG2 (right) cells under indicated PA concentrations for 72 hours as determined by MTT analysis. Data shown as mean ± SD. * p < 0.05; ** 0.001 < p < 0.01; *** p < 0.001.


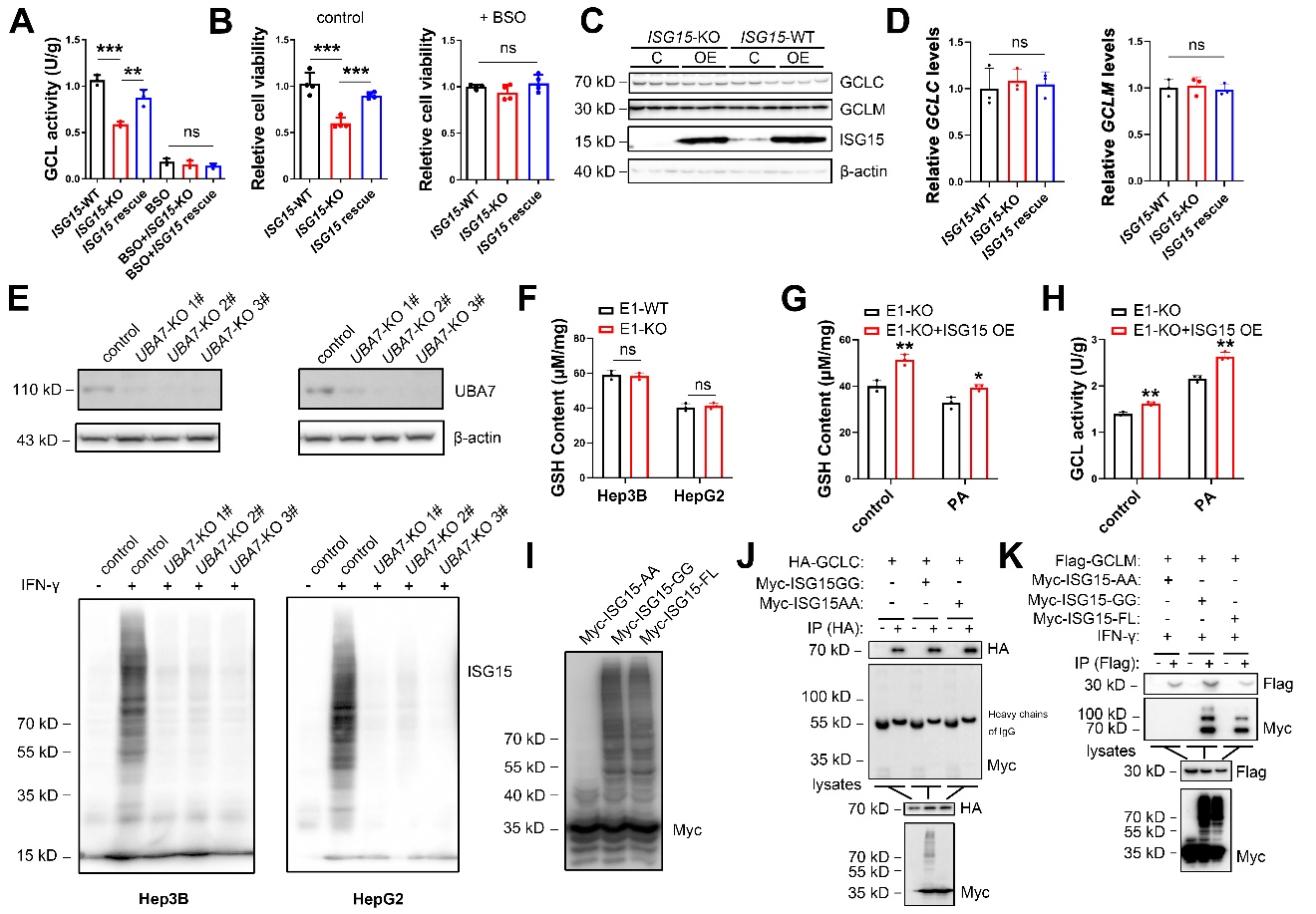


**Figure S3. ISG15 promotes glutathione production in an ISGylation independent manner.** (**A**) γ-glutamate cysteine ligase activity in *ISG15*-WT/KO and ISG15-rescued Hep3B cells under 300 μM of PA treatment for 24 hours with or without BSO treatment (10 μM). (**B**) Viability of Hep3B cells under 300 μM of PA treatment for 72 hours with or without BSO treatment (10 μM) as determined by MTT analysis. (**C**) Western blots of GCLC and GCLM in *ISG15*-WT/KO Hep3B cells overexpressing foreign ISG15 (OE). (**D**) mRNA levels of *GCLC* and *GCLM* in *ISG15*-WT/KO and ISG15-rescued Hep3B cells. (**E**) Western blots of endogenous UBA7 in *UBA7*-WT and *UBA7*-KO Hep3B and HepG2 cells (Top) and western blots of ISGylation in IFN-γ treated *UBA7*-WT and *UBA7*-KO Hep3B and HepG2 cells (Bottom). (**F**) Total glutathione levels in *UBA7*-WT and *UBA7*-KO HepG2 and Hep3B cells. (**G**) Total glutathione levels in *UBA7*-KO HepG2 cells overexpressing ISG15 with or without 300 μM of PA treatment. (**H**) γ-glutamate cysteine ligase activities in *UBA7*-KO HepG2 cells overexpressing ISG15 with or without 300 μM of PA treatment. (**I**) Western blots of ISGylation in Hep3B cells overexpressing ISG15-GG, ISG15-AA and ISG15 full length. (**J**) 293T cells co-transfected with HA-GCLC and/or Myc-ISG15-GG/AA as indicated. Anti-HA immunoprecipitates (top) and total lysates (bottom) were subject to immunoblot with anti-HA antibody to reveal foreign GCLC, and anti-Myc antibody to reveal foreign ISG15. (**K**) Co-immunoprecipitation of GCLM and ISG15. 293T cells co-transfected with Flag-GCLM and Myc-ISG15-GG/AA as indicated, anti-Flag immunoprecipitates (top) and total lysates (bottom) were subjected to immunoblot with anti-Flag antibody to reveal foreign GCLM, and anti-Myc antibody to reveal ISGylated GCLM (about 70 kD and 100 kD). Data shown as mean ± SD. ns, not significant; * p < 0.05; ** p < 0.01. *** p < 0.001.


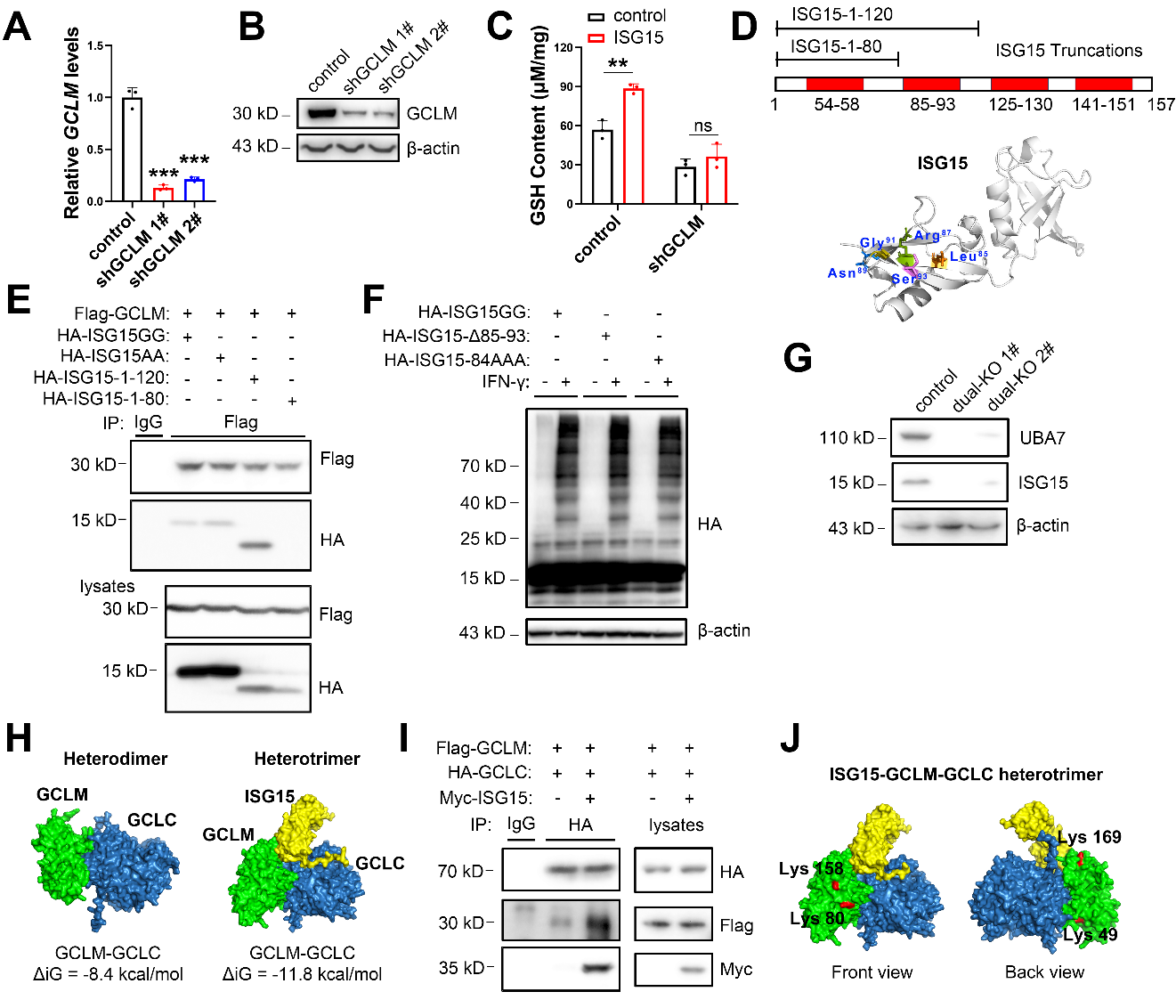


**Figure S4. ISG15 promotes GCLC-GCLM interaction through binding with GCLM.** (**A**) mRNA levels of *GCLM* in *GCLM*-knockdown Hep3B cells. (**B**) Western blots of GCLM in *GCLM*-knockdown Hep3B cells. (**C**) Glutathione levels in *GCLM*-KD Hep3B cells overexpressing ISG15. (**D**) ISG15 truncation constructs. The potential binding regions on ISG15 with GCLM are indicated. Representative structure of ISG15 with residues 85-93 is indicated. (**E**) Co-immunoprecipitation between GCLM and indicated ISG15 constructs. 293T cells were co-transfected with Flag-GCLM, and HA-ISG15 truncation constructs as indicated. Anti-Flag immunoprecipitates (top) and total lysates (bottom) were subjected to immunoblot with anti-Flag antibody to reveal foreign GCLM, and anti-HA antibody to reveal foreign ISG15 constructs. (**F**) Western blots of ISGylation in Hep3B cells overexpressing ISG15 mutants treated with IFN-γ. (**G**) Western blots of endogenous UBA7 and ISG15 in *ISG15/UBA7* dual-KO Hep3B cells. (**H**) Representative structure of GCLM-GCLC dimer and ISG15-GCLM-GCLC complex. The delta-iG of GCLM/GCLC is indicated. (**I**) Co-immunoprecipitation between GCLM/GCLC and ISG15. *ISG15*-KO Hep3B cells were co-transfected with Flag-GCLM, HA-GCLC and Myc-ISG15 as indicated. Anti-HA immunoprecipitates (left) and total lysates (right) were subjected to immunoblot with anti-Flag antibody to reveal foreign GCLM, anti-HA antibody to reveal foreign GCLC, and anti-Myc antibody to reveal foreign ISG15. (**J**) Representative structure of an ISG15-GCLM-GCLC complex. Potential ISGylation sites on GCLM are indicated. Data shown as mean ± SD. ns, not significant; * p < 0.05; ** p < 0.01. *** p < 0.001.


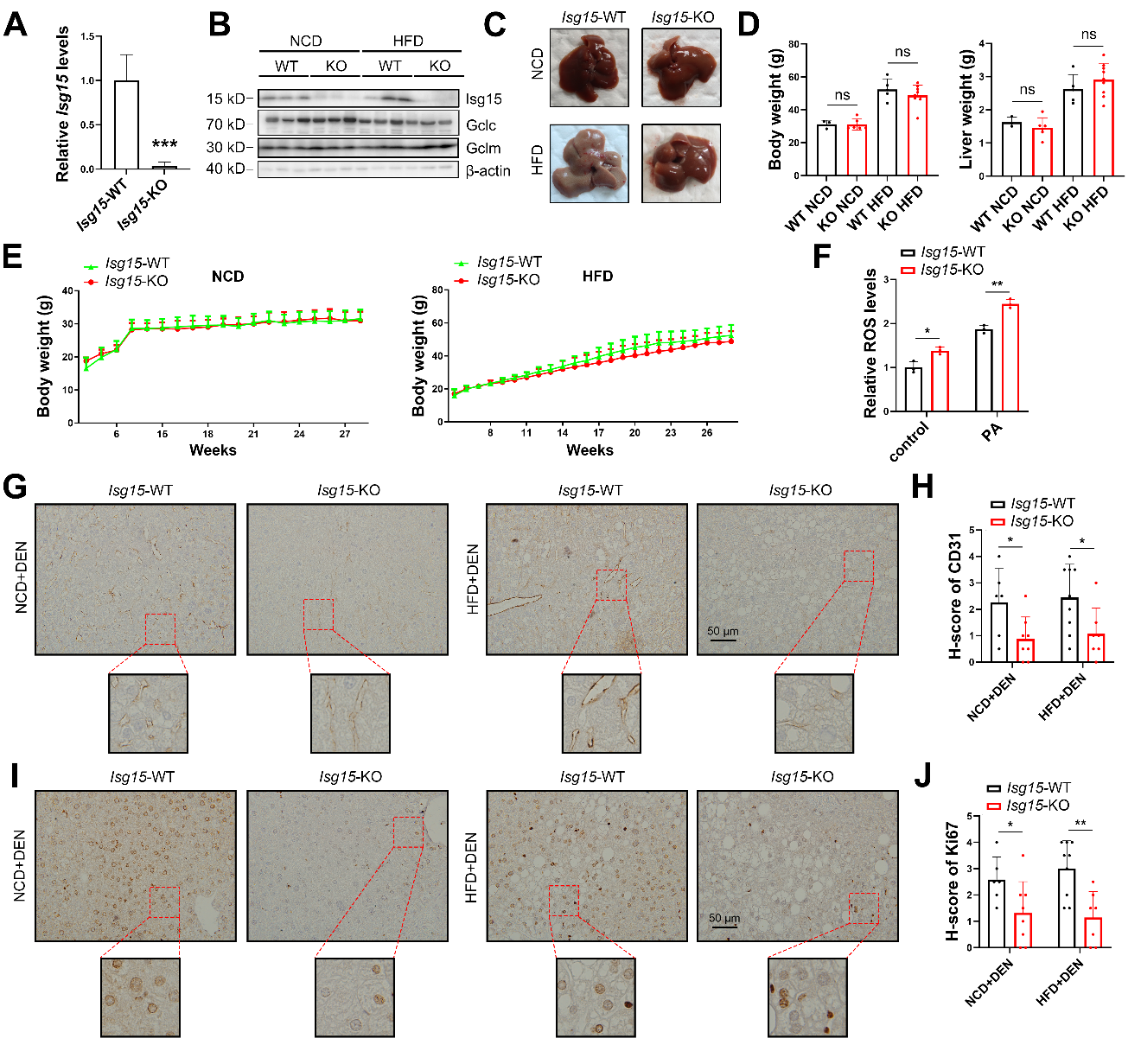


**Figure S5. Ablation of *Isg15* does not affect simple hepatic steatosis.** (**A**) mRNA levels of *Isg15* in liver from *Isg15*-WT and *Isg15*-KO mice. (**B**) Western blots of liver from NCD- or HFD-fed *Isg15*-WT and *Isg15*-KO mice. (**C**) Representative images of livers from NCD- or HFD-fed *Isg15*-WT and *Isg15*-KO mice. (**D**) Body and liver weight of *Isg15*-WT and *Isg15*-KO mice fed with NCD or HFD diets for 28 weeks. (**E**) Body weight curve of *Isg15*-WT and *Isg15*-KO mice fed with NCD or HFD diets for 28 weeks. (**F**) ROS levels of primary liver cells from *Isg15*-WT/KO mice with or without PA treatments determined by FACS analysis. (**G-H**) Representative images (**G**) and H-score quantification (**H**) of CD31 IHC staining in murine HCC tissues. (**I-J**) Representative images (**I**) and H-score quantification (**J**) of Ki67 IHC staining in murine HCC tissues. Data shown as mean ± SD. ns, not significant. * p < 0.05; ** p < 0.01. *** p < 0.001.

**Supplementary Table 1. Antibody information.**

| **Antibody** | **Catalog number** | **Vendor** |
| --- | --- | --- |
| ISG15 (h) | No. 15981-1-AP | Proteintech |
| Isg15 (m) | PA5-79523 | Invitrogen |
| Tubulin | sc-166729 | Santa Cruz |
| HMGA1 | sc-8982 | Santa Cruz |
| IRF-3 | HY-P80504 | MCE |
| p-IRF-3 | bs-3195R | Bioss |
| Caspase-9 | ab202068 | Abcam |
| Caspase-8 | 13423-1-AP | Proteintech |
| Caspase-3 | #9661 | Cell Signaling Technology |
| β-actin | sc-8432 | Santa Cruz |
| Flag | F1804 | Sigma Aldrich |
| HA | H9658 | Sigma Aldrich |
| Myc | 16286-1-AP | Proteintech |
| GCLM | No. 14241-1-AP | Proteintech |
| GCLC | No. 12601-1-AP | Proteintech |
| UBA-7 | abs146504 | Absin |
| CD31 | #77699 | Cell Signaling Technology |
| Ki67 | ab66155 | Abcam |

h, human; m, mouse

**Supplementary Table 2. Target sequences for gRNA and shRNA.**

| **Target** | **Sequence** |
| --- | --- |
| ISG15 (gRNA) | TTGAGGCCGTACTCCCCCAG |
| UBA7 (gRNA) | CGCACTAGGGCCTCATCCAG |
| HMGA1 (gRNA) | ACCCGGGTGAGACTTGAGAT |
| GCLM (shRNA) | GCTGGATTCTGTGATCATT |

**Supplementary Table 3. qPCR primers.**

| **Gene** | **Forward** | **Reverse** |
| --- | --- | --- |
| *ISG15* | CTCTGAGCATCCTGGTGAGGAA | AAGGTCAGCCAGAACAGGTCGT |
| *Isg15* | CATCCTGGTGAGGAACGAAAGG | CTCAGCCAGAACTGGTCTTCGT |
| *β-actin* | CCAAGGCCAACCGCGAGAAGATGAC | AGGGTACATGGTGGTGCCGCCAGAC |
| *Irf-3* | CGGAAAGAAGTGTTGCGGTTAGC | CAGGCTGCTTTTGCCATTGGTG |
| *IRF-3* | TCTGCCCTCAACCGCAAAGAAG | TACTGCCTCCACCATTGGTGTC |
| *Hmga1* | AGTGCCAACTCCGAAGAGACCT | TTGGTTTCCTCCCTGGAGCTGT |
| *HMGA1* | GAAGTGCCAACACCTAAGAGACC | GGTTTCCTTCCTGGAGTTGTGG |
| *Gclm* | TCCTGCTGTGTGATGCCACCAG | GCTTCCTGGAAACTTGCCTCAG |
| *GCLM* | TCTTGCCTCCTGCTGTGTGATG | TTGGAAACTTGCTTCAGAAAGCAG |
| *Gclc* | ACACCTGGATGATGCCAACGAG | CCTCCATTGGTCGGAACTCTAC |
| *GCLC* | GGAAGTGGATGTGGACACCAGA | GCTTGTAGTCAGGATGGTTTGCG |
| *UBA7* | GAACTCTTCCGACTGTCTGCAG | AGTCTTGCCAGTTCTGTGGACG |

**Supplementary Table 4. Primer sequences for ChIP and reporter assays.**

| **Target** | **Forward** | **Reverse** |
| --- | --- | --- |
| ISG15 P1 | ACTGCCCTAAACCGAGTGTT | ATCGCGCATTCCAGATCCTT |
| ISG15 P2 | TATGTCCTGGATGCTATGC | CTGCCTCGGCCTCTGAAACTG |
| ISG15 P3 | CCCGCCTCAGCCTCCCAAGTAG | CTCACGCCTGCAATCCCAACAC |
| ISG15 P4 | ACGGGGTCGGCTTCCTGTGC | GCCGCCGCGATGCTCAAA |
| ISG15 promoter | AATCTCGAGTTGTCCAACCATCAC | ATTAAGCTTCGGCTTCGGCAGGCA |

**Supplementary Table 5. Fold changes of candidate transcription factors/cofactors of *Isg15* between HFD- and NCD-fed mice.**

| **Candidate**  **Transcription factors/cofactors** | **Fold change**  **(HFD/NCD)** | **p value** |
| --- | --- | --- |
| *Foxa1* | 0.70 | p > 0.05 |
| *Irf1* | 0.94 | p > 0.05 |
| *Irf2* | 1.08 | p > 0.05 |
| *Irf3* | 0.73 | p = 0.027 |
| *Irf4* | not detected | - |
| *Klf4* | 1.58 | p > 0.05 |
| *Nfat5* | 1.01 | p > 0.05 |
| *Stat2* | 1.23 | p > 0.05 |
| *Vdr* | not detected | - |
| *Hmga1* | 1.69 | p = 0.012 |

**Supplementary Table 6. Potential interacting residues on GCLM/GCLC/ISG15 surfaces simulated by Alphafold2 PPI modeling.**

|  | **ISG15** | **GCLM** | **GCLC** |
| --- | --- | --- | --- |
| **ISG15** | - | 54-58, 85-93, 125-130, 141-151 | 55-59, 85-90, 123-131, 142-154 |
| **GCLM** | 29-41, 189-198, 213-217 | - | 24-44, 84-93, 122-127, 187-198, 209-216, 249-264 |
| **GCLC** | 41-46, 86-90, 107-117, 255-259, 449-457, 497-502, 541-548 | 41-47, 111-115, 256-261, 450-458, 488-500, 532-556, 623-626 | - |

**Supplementary Table 7. Binding energies of GCLM/GCLC/ISG15 complexes calculated by Alphafold2 PPI modeling.**

| **Complex** | **Intermolecular interaction** | **Δ^i^G**  **(kcal/mol)** |
| --- | --- | --- |
| **GCLM-GCLC** | GCLM-GCLC | -8.4 |
| **ISG15-GCLM-GCLC** | GCLM-GCLC | -11.8 |
| **ISG15-GCLM** | ISG15-GCLM | -3.9 |
| **ISG15-GCLC** | ISG15-GCLC | -1.9 |

**Supplementary Table 8. Potential ISGylation sites on GCLM.**

|  | **10**  **upstream residues** | **10**  **downstream residues** | **ISG15**  **interfacing residues** | **GCLC**  **interfacing residues** |
| --- | --- | --- | --- | --- |
| **K49** | HSEELHDCIQ | TLNEWSSQIN | no | no |
| **K80** | LECTVSHAVE | INPDEREEMK | no | no |
| **K158** | EELENLVQSK | IVAIGTSDLD | no | no |
| **K169** | IVAIGTSDLD | TQLEQLYQWA | no | no |

The sites were predicted according to the study by Zhu, *et al.*, Vaccines (Basel), 2021.
